# Synthesis and Evaluation of ^99m^Tc-Labelled 2-Nitroimidazole Derivatives with Different Linkers for Tumour Hypoxia Imaging

**DOI:** 10.3390/ph16091276

**Published:** 2023-09-09

**Authors:** Qing Ruan, Yitong Liu, Lihao Liao, Jinyu Hao, Yuhao Jiang, Jianyong Jiang, Junbo Zhang

**Affiliations:** 1 Key Laboratory of Beam Technology of the Ministry of Education, College of Nuclear Science and Technology, Beijing Normal University, Beijing 100875, China; 11132022029@bnu.edu.cn; 2 Key Laboratory of Radiopharmaceuticals of the Ministry of Education, NMPA Key Laboratory for Research and Evaluation of Radiopharmaceuticals (National Medical Products Administration), College of Chemistry, Beijing Normal University, Beijing 100875, China; 202011150006@mail.bnu.edu.cn (Y.L.); 201911081124@mail.bnu.edu.cn (L.L.); 202011150010@mail.bnu.edu.cn (J.H.); 202131150056@mail.bnu.edu.cn (Y.J.)

**Keywords:** tumour hypoxia imaging, 2-nitroimidazole, linker, SPECT/CT, ^99m^Tc

## Abstract

When developing novel radiopharmaceuticals, a linker moiety between the chelator and targeting vector can have a crucial influence on adjusting the affinity of the tracer and its biodistribution in organisms. To develop novel ^99m^Tc-labelled hypoxia imaging radiotracers, in this study, five isocyanide-containing 2-nitroimidazole derivatives with different linkers (L1, L2, L3, L4 and L5) were synthesised and radiolabelled with technetium-99m to obtain five stable ^99m^Tc-complexes ([^99m^Tc]Tc-L1, [^99m^Tc]Tc-L2, [^99m^Tc]Tc-L3, [^99m^Tc]Tc-L4 and [^99m^Tc]Tc-L5). Corresponding rhenium analogues of [^99m^Tc]Tc-L1 were synthesised and suggested the structures of these ^99m^Tc-complexes would be a monovalent cation with a technetium (I) core surrounded by six ligands. [^99m^Tc]Tc-L1 is hydrophilic, while the lipophilicities of [^99m^Tc]Tc-L2, [^99m^Tc]Tc-L3, [^99m^Tc]Tc-L4 and [^99m^Tc]Tc-L5 are close. In vitro cell experiments showed that all five novel ^99m^Tc-complexes had higher uptake in hypoxic cells compared with aerobic cells, which indicates the complexes have good hypoxia selectivity. The biodistribution of the five ^99m^Tc-complexes in S180 tumour-bearing mice showed that they all had certain uptake in the tumours. Among them, [^99m^Tc]Tc-L1 had the highest tumour-to-muscle (4.68 ± 0.44) and tumour-to-blood (3.81 ± 0.46) ratios. The introduction of polyethylene glycol (PEG) chains effectively reduced the lipophilicity and decreased uptake by the liver, intestine and blood but also increased clearance from the tumours. In vivo metabolic studies showed [^99m^Tc]Tc-L1 kept intact and remained stable in tumour, blood and urine at 2 h post-injection. The results of SPECT imaging showed that [^99m^Tc]Tc-L1 had significant tumour uptake at 2 h post-injection, but there was still high uptake in abdominal organs such as the liver and kidney, suggesting that this complex needs to be further optimised before being used for tumour hypoxia imaging.

## 1. Introduction

The oxygen content in the majority of tumour tissues is notably lower than that in other tissues due to the uncontrollable growth of tumour cells that consume more oxygen than normal tissues [1,2]. Additionally, disordered growth of neovascularisation also leads to the development of chronic diffusion-limited hypoxia [3,4]. Increased pressure on immature blood vessels and tumour stroma can also lead to vascular collapse, which is the cause of acute or intermittent hypoxia. Tumour hypoxia may result in resistance to chemotherapy and radiotherapy and an increased possibility of metastasis [2]. Therefore, tumour hypoxia is an important characteristic of the tumour microenvironment and plays an important role in clinical treatment evaluation [5,6]. Tumour hypoxia not only has a direct impact on tumour therapy but also has been found to be related to many molecular signalling pathways that affect tumour manifestation. The transcriptional regulation of hypoxia-inducible genes has been widely recognised since the discovery of hypoxia-inducible factor (HIF). Thus, it is of great significance to know the oxygen state in tumours [7,8,9].

2-Nitroimidazole can be reduced by nitroreductase in cells, and the intermediate products bind irreversibly around the cells in the hypoxic region, effectively marking the hypoxic region of the tumour. The mechanism of nitroimidazole targeting hypoxic cells is that, after the molecules enter the cells, the nitro group on the nitroimidazole undergoes one-electron reduction under the actions of nitroreductase and generates free radical anions. In cells with a normal oxygen content, this molecule is oxidised back to the original compound and diffuses extracellularly; while in hypoxic cells, the molecule is further reduced and retained in the cell by binding to cellular macromolecules [7]. 2-Nitroimidazole derivatives are more suitable for the detection of tumour hypoxia because these compounds have a more positive single-electron reduction potential (SERP) value than 4-nitroimidazole and 5-nitroimidazole, which means it can be efficiently reduced and remain in hypoxic tissue [10]. Tumour hypoxia imaging agents have been studied for many years, and several radiotracers with 2-nitroimidazole derivatives have been reported [11,12,13,14]. [^18^F]fluoromisonidazole ([^18^F]FMISO, 1H-1-(3-[^18^F]fluoro-2-hydroxypropyl)-2-nitroimidazole) is a widely used tumour hypoxia imaging agent in clinic PET imaging [14]. However, it still has the disadvantages of a slow clearance rate from aerobic tissues and a low tumour-to-muscle ratio [15]. In addition, the preparation of ^18^F requires a cyclotron, which also limits the use of [^18^F]FMISO. Compared with ^18^F, ^99m^Tc has an ideal half-life, is easy to prepare with a ^99^Mo-^99m^Tc generator and comes with a reasonable cost [16]. Thus, ^99m^Tc has become the most widely used SPECT radioisotope, and there are a large number of SPECT scanners worldwide. Previously reported ^99m^Tc-labelled 2-nitroimidazole derivative imaging agents, such as BMS-181321 [17], [^99m^Tc]Tc(NS_3_) [18], [^99m^Tc]Tc-4 [19] and [^99m^Tc]Tc-2c [20], all showed selectivity for hypoxia and moderate tumour uptake but still had the disadvantage of high uptake in nontarget tissues, such as the blood, liver, intestines and kidneys. Ideal ^99m^Tc-labelled tumour hypoxia imaging agents are still in great demand [21].

For isonitrile complexes, the nitrogen atom has a partial positive charge, while the carbon atom has a partial negative charge, which has a strong electron-donating effect. Therefore, isonitrile compounds, as a kind of monodentate ligand, have good coordination ability with the ^99m^Tc core to produce stable [^99m^Tc][Tc(CN-R)_6_]^+^ complexes with six ligands [20,22,23]. Linker molecules have the effects of adjusting the hydrophilicity and the biodistribution properties of tracers [24]. To improve tumour uptake of most tumour hypoxia imaging agents and reduce their retention in blood and uptake in liver, intestines and kidneys, in this study, five novel 2-nitroimidazole isocyanide derivatives with different linkers were designed, synthesised and coordinated with ^99m^Tc to form five novel ^99m^Tc-labelled complexes. These ^99m^Tc-labelled complexes were tested by partition coefficient, in vitro stability, in vitro cellular uptake, biodistribution and SPECT/CT imaging studies to evaluate their potential as tumour hypoxia imaging agents.

## 2. Results

### 2.1. Chemistry

**Scheme 1:**
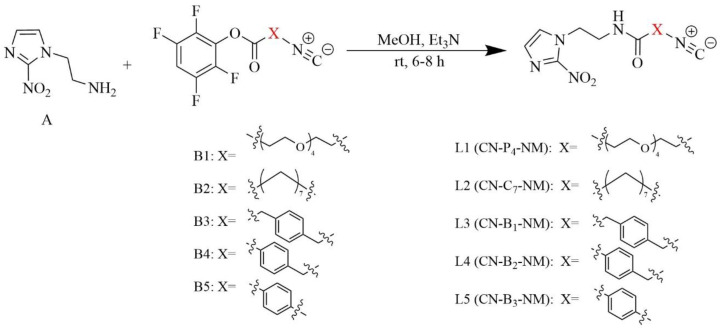
The synthesis of ligands (**L1–L5**).

The 2-nitroimidazole amino derivative (Compound **A**) was synthesised from 2-nitroimidazole in two steps, while the isocyanide-containing active esters (Compounds **B1–B5**) were synthesised in three steps; the details of these syntheses are exhibited in the Supplementary Data. The ligands **L1–L5** were synthesised with Compound **A** and Compounds **B1–B5**, as shown in Scheme 1, and identified by ^1^H-NMR, ^13^C-NMR, IR and HR-MS. Characterisation data demonstrate that the target ligands had been synthesised.

### 2.2. Radiolabelling and Quality Control

**Figure 1:**
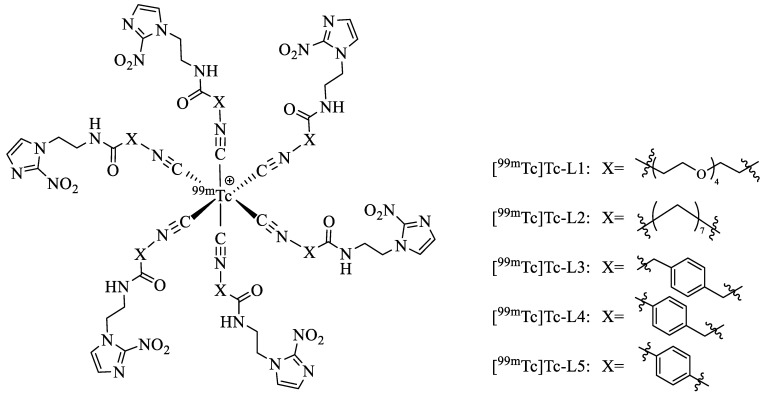
Predicted chemical structures of the ^99m^Tc-complexes.

**Figure 2:**
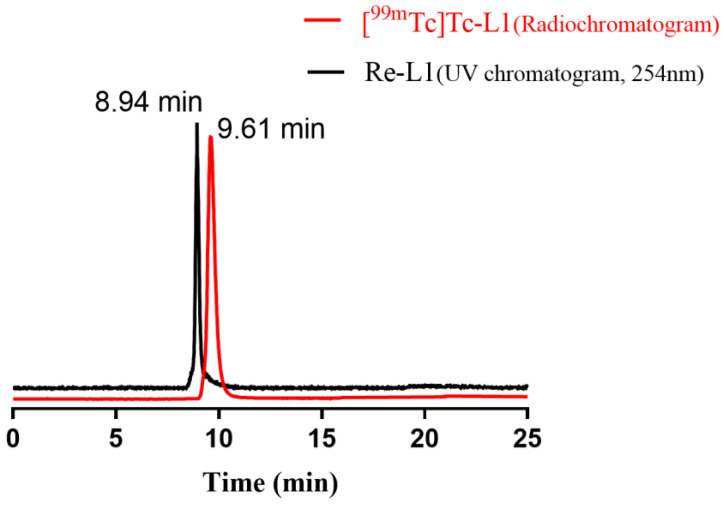
Co-injection analysis of [^99m^Tc]Tc-L1 (radiochromatogram) and Re-L1 (UV chromatogram, 254 nm).

By reaction of pertechnetate with the reducing agent, stannous chloride, provided in the kit, the ^99m^Tc-complexes can be prepared in 20 min at 100 °C in one pot. The radiochemical purity (RCP) was over 90% with small percentages of [^99m^Tc]Tc-citrate and pertechnetate as assessed by thin layer chromatography (TLC) and high-performance liquid chromatography (HPLC), suggesting that in vitro and in vivo studies can be carried out without further purification. The proposed structure of these complexes is monovalent cation with a ^99m^Tc core in the centre surrounded by six identical ligands, which are similar to that of [^99m^Tc]Tc-sestamibi (sestamibi: 2-methoxy-2-isobutyl isonitrile). The predicted chemical structures of the five complexes are shown in Figure 1. The corresponding rhenium analogues of [^99m^Tc]Tc-L1 were synthesised and identified by ^1^H-NMR and MS to verify the structures of the ^99m^Tc-complexes, as both Tc and Re belong to the ⅦB group and have similar chemical properties. The HPLC profile of [^99m^Tc]Tc-L1 co-injected with Re-L1 is shown in Figure 2. The radioactivity curve of [^99m^Tc]Tc-L1 (t_R_ = 9.61 min) was close to the UV pattern of Re-L1 (t_R_ = 8.94 min), indicating that [^99m^Tc]Tc-L1 had the proposed structure.

### 2.3. In Vitro Stability Study

**Figure 3:**
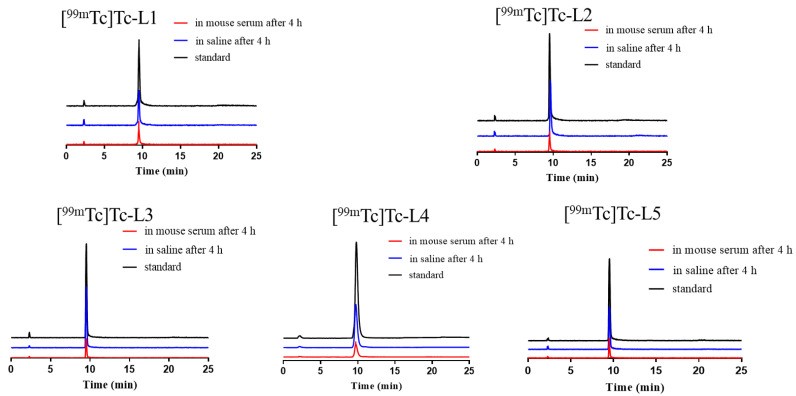
HPLC results of the ^99m^Tc-complexes. After radiolabelling (black); stability in saline at r.t. after 4 h (blue) and stability in mouse serum at 37 °C after 4 h (red).

The RCP of these complexes can be used to evaluate the stability of the complexes. As shown in Figure 3, after the five ^99m^Tc-labelled complexes were kept in saline at r.t. or in mouse serum at 37 °C for 4 h, the RCPs of them were all higher than 90%, suggesting that these complexes do not decompose within 4 h and have good stability in vitro.

### 2.4. Determination of the Partition Coefficients of the Complexes

The Log D values of the five tracers are shown in Table 1, and the data indicate that [^99m^Tc]Tc-L1 is hydrophilic, while the lipophilicities of [^99m^Tc]Tc-L2, [^99m^Tc]Tc-L3, [^99m^Tc]Tc-L4 and [^99m^Tc]Tc-L5 are close. Compared with [^99m^Tc]Tc-L2, which contains a carbon chain (n = 7), [^99m^Tc]Tc-L1 containing a polyethylene glycol (PEG, n = 4) chain has a lower Log D value, indicating that [^99m^Tc]Tc-L1 is more hydrophilic and the addition of PEG chains can effectively improve the hydrophilicity. By comparing [^99m^Tc]Tc-L3, [^99m^Tc]Tc-L4 and [^99m^Tc]Tc-L5, it was found that the lipophilicities of the three ^99m^Tc-complexes with phenyl ring were close, indicating that there was no positive correlation between the increase in the number of carbon atoms on both sides of the phenyl ring in these substituents and the lipophilicity of these ^99m^Tc-complexes.

### 2.5. In Vitro Cellular Uptake Study

**Figure 4:**
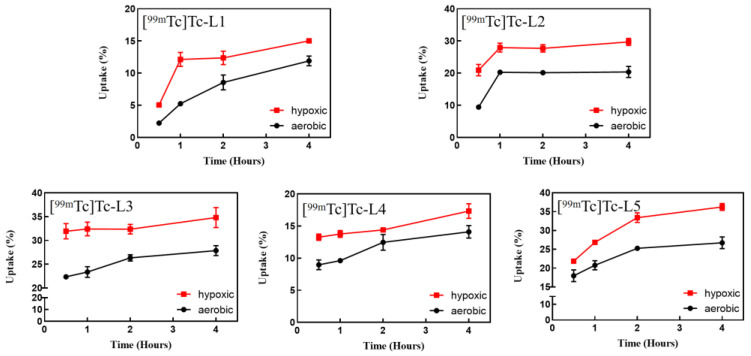
In vitro cellular uptake of the studied ^99m^Tc-complexes in S180 cells (hypoxia vs. normoxia, *p* < 0.05).

The in vitro cellular uptake of five tracers was tested on S180 cells under both aerobic and hypoxic conditions. Figure 4 shows that the uptake of the five complexes under hypoxic conditions was significantly higher than the uptake under aerobic conditions (*p* < 0.05, in Table S1), which indicates that they had good hypoxia selectivity.

### 2.6. Biodistribution Studies

**Figure 5:**
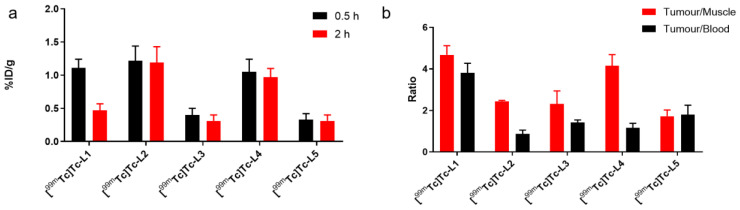
Tumour uptake at 0.5 h and 2 h post-injection (**a**) and tumour-to-blood and tumour-to-muscle ratios at 2 h post-injection (**b**) of ^99m^Tc-complexes (n = 5).

The results of biodistribution studies of the ^99m^Tc-complexes are shown in Table 2. From Figure 5a, it is obvious that among these five isonitrile nitroimidazole complexes containing different linkers, [^99m^Tc]Tc-L2, containing a carbon chain (n = 7), had the highest tumour uptake value (1.22 ± 0.22%ID/g) and good retention in tumours for 2 h (1.19 ± 0.24%ID/g). However, the high uptake of [^99m^Tc]Tc-L2 in the liver (33.31 ± 3.75%ID/g) and kidney (24.70 ± 2.77%ID/g) may increase the difficulty in the detection of abdominal tumour. By comparing the ^99m^Tc-complexes containing phenyl rings ([^99m^Tc]Tc-L3, [^99m^Tc]Tc-L4 and [^99m^Tc]Tc-L5), it was found that [^99m^Tc]Tc-L4 had the highest tumour uptake and good retention, with an uptake of 0.97 ± 0.13%ID/g at 2 h, and the highest tumour/muscle ratio, which reached 4.15 ± 0.54 at 2 h post-injection. Among these three complexes containing phenyl rings as linkers, uptake in the liver and spleen increased with the reduced numbers of carbon atoms. For [^99m^Tc]Tc-L5, the uptake in the liver at 0.5 h was 45.08 ± 8.90% ID/g and the uptake in the spleen was 36.16 ± 3.80%ID/g. In fact, these ^99m^Tc-complexes containing phenyl rings are not ideal tumour hypoxia imaging agents because of their slow clearance in vivo and high uptake in nontarget organs, including the liver, kidneys and spleen. In comparison, [^99m^Tc]Tc-L1 containing tetra-oxyethylene PEG chains had the highest tumour-to-muscle (4.68 ± 0.44) and tumour-to-blood ratios (3.81 ± 0.46), and the ratios of the tumour to other nontarget tissues were significantly reduced, indicating that [^99m^Tc]Tc-L1 was more favourable than the other four ^99m^Tc-complexes for further research.

### 2.7. SPECT/CT Imaging Studies

**Figure 6:**
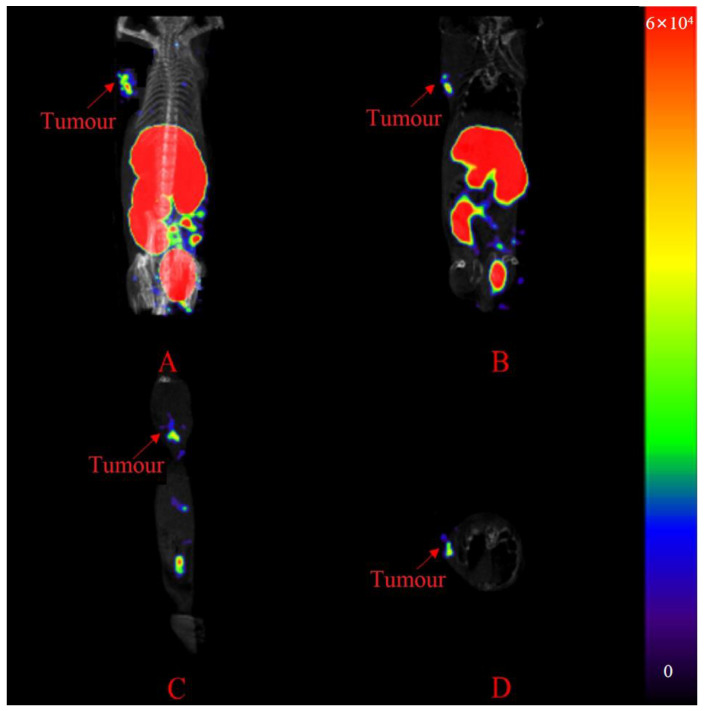
SPECT/CT images of [^99m^Tc]Tc-L1 at 2 h post-injection. Maximum intensity projection (**A**), sagittal section (**B**), coronal section (**C**) and transverse section (**D**).

[^99m^Tc]Tc-L1 was selected to conduct SPECT/CT imaging studies because of its high target-to-nontarget ratios. SPECT/CT images are shown in Figure 6. In the left front axilla of the mice, the tumours were clear and distinct at 2 h post-injection, but the uptake by the liver and kidneys was still obvious. SPECT images of [^99m^Tc]Tc-L1 at 0.5 h, 1 h and 2 h post-injection are shown in Figure S18.

### 2.8. In Vivo Metabolic Studies

**Figure 7:**
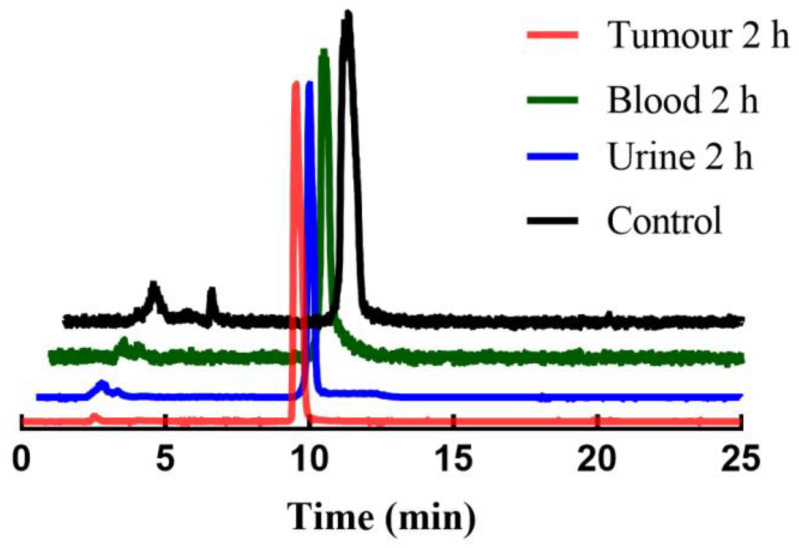
HPLC profiles of the radioactivity in tumour, blood and urine of S180 tumour-bearing mice at 2 h post-injection of [^99m^Tc]Tc-L1.

To investigate the in vivo metabolism of [^99m^Tc]Tc-L1, S180 tumour-bearing mice were used for metabolic studies in tumour, blood, and urine at 2 h post-injection. As shown in Figure 7, there is clearly some (but limited) degradation of the tracer in blood, urine and tumour, but it mainly kept intact (>90%) and remained stable in vivo.

## 3. Discussion

Tumour hypoxia can increase the resistance of tumours to radiotherapy and chemotherapy and increase the aggressiveness of the tumour itself [25,26]. Therefore, detecting tumour hypoxia is important for formulating treatment plans and improving treatment effects.

There are many methods by which tumour hypoxia can be diagnosed, such as oxygen measurement using Eppendorf and optical needle probes [27,28]. Nuclear medicine imaging based on SPECT and PET can noninvasively detect the degree of tumour hypoxia in vivo, and this method is simple and feasible. It also provides a basis for diagnosis, staging, efficacy detection and prognosis evaluations of tumours [7]. Compared with PET, SPECT has a wider range of radionuclide sources and a lower cost, especially for ^99m^Tc, making it the preferred method in nondeveloped regions. In addition, the resolution and sensitivity of SPECT devices have undergone great development due to the use of cadmium zinc telluride (CZT) crystals [29]. Therefore, it is necessary to develop SPECT tumour imaging agents with excellent performance to meet clinical needs. Since ^99m^Tc has been the most widely used SPECT radionuclide, accounting for more than 70% of the annual diagnostic radiopharmaceuticals in clinics [30,31], a ^99m^Tc-labelled radiotracer has been a focal point of research in this work.

As linkers can significantly alter the biodistribution potency of compounds, the purpose of this study was to design, synthesise and evaluate novel ^99m^Tc-labelled 2-nitroimidazole derivatives with modified linkers as hypoxia imaging agents. Five isocyanide-containing 2-nitroimidazole derivatives with different linkers (L1, L2, L3, L4 and L5) were synthesised and identified by ^1^H-NMR, ^13^C-NMR, IR and HR-MS. Isocyanide (CN-R) is regarded as a kind of monodentate ligand that can strongly coordinate with the ^99m^Tc core, thus the ligands could be conveniently radiolabelled with technetium-99m using a kit formulation in a one-pot reaction to obtain five ^99m^Tc-complexes ([^99m^Tc]Tc-L1, [^99m^Tc]Tc-L2, [^99m^Tc]Tc-L3, [^99m^Tc]Tc-L4 and [^99m^Tc]Tc-L5). It should be noted that the preparations of these ^99m^Tc-complexes require heating at 100 °C. The structural characterisation of ^99m^Tc-complexes is difficult at the no-carrier-added level, but because Tc and Re are congeners and they have similar chemistry properties, corresponding rhenium analogues can be synthesised at the macroscopic level. The corresponding rhenium analogues of [^99m^Tc]Tc-L1 were synthesised and identified by ^1^H-NMR and MS to confirm that the structure of the ^99m^Tc-complexes is a monovalent cation with a ^99m^Tc core in the centre surrounded by six identical ligands. The five ^99m^Tc-complexes all exhibited excellent in vitro stability. The Log D values of the five ^99m^Tc-complexes showed that [^99m^Tc]Tc-L1 had the highest hydrophilicity. In vitro cell experiments showed that all five novel ^99m^Tc-complexes had higher uptake in hypoxic cells compared with aerobic cells, which indicates the complexes have favourable hypoxia selectivity. Female Kunming mice bearing S180 tumours, which were used as a model of solid tumour with hypoxia [32,33], were used for the biodistribution study. The tumour uptake value of [^99m^Tc]Tc-L1 (1.11 ± 0.13% ID/g) containing a PEG chain was slightly lower than that of [^99m^Tc]Tc-L2. However, the tumour retention of [^99m^Tc]Tc-L1 at 2 h post-injection was only 40% of that at 0.5 h post-injection, and the fast clearance from the tumour might relate to its hydrophilicity. The introduction of the PEG chain effectively reduced the lipophilicity and decreased the uptake in the liver, intestine and blood, but it also accelerated the clearance from the tumours [34,35]. Interestingly, due to its rapid clearance from nontarget organs, [^99m^Tc]Tc-L1 had the highest tumour/muscle (4.68 ± 0.44) and tumour/blood (3.81 ± 0.46) ratios, indicating the potential of this compound for further research. To investigate clearance in vivo, we studied the biodistribution at 6 h post-injection of [^99m^Tc]Tc-L1. With faster clearance in kidneys and intestine and some retention of the tracer in tumour, [^99m^Tc]Tc-L1 shows a preferable target-to-nontarget ratio. Study of the in vivo metabolism of [^99m^Tc]Tc-L1 was carried out by analysis of the radioactivity in different tissues using HPLC. The results showed that the tracer remained mostly intact and thus was stable in vivo. Only small percentages of metabolites were found in blood, urine and tumour. 

In previous work, ^99m^Tc-2c, a ^99m^Tc-labelled 2-nitroimidazole derivative containing a carbon chain (n = 4), was reported to have a good tumour-to-muscle ratio (5.05) [20]. Compared with ^99m^Tc-2c, [^99m^Tc]Tc-L1 containing a PEG chain as the linker showed a lower retention in blood and exhibited for this reason a higher tumour-to-blood ratio at 2 h post-injection (T/B for [^99m^Tc]Tc-L1: 3.81 vs. T/B for ^99m^Tc-2c: 1.32). Compared with the cyclopentadienyl tricarbonyl technetium-99m 2-nitroimidazole derivative [^99m^Tc]Tc-4, [^99m^Tc]Tc-L1 showed higher tumour-to-muscle ratio (T/M for [^99m^Tc]Tc-L1:4.68 vs. T/M for [^99m^Tc]Tc-4: 3.47) and tumour-to-blood ratio (T/B for [^99m^Tc]Tc-L1:3.81 vs. T/B for [^99m^Tc]Tc-4: 0.59) [19] at 2 h post-injection. 

As [^99m^Tc]Tc-L1 had higher tumour-to-blood and tumour-to-muscle ratios than the other four tracers, it was chosen as a prospective tracer for further SPECT/CT imaging studies. The SPECT/CT imaging results of [^99m^Tc]Tc-L1 exhibited observable tumour uptake, which was consistent with the results of biodistribution studies in mice. However, the relatively high uptake of [^99m^Tc]Tc-L1 in the liver and kidneys is a shortcoming of the complex, and the results of abdominal tumour imaging may be affected. In future studies, we will focus on improving the tumour uptake and retention of hypoxic tumour molecular probes and reducing their uptake in the liver and kidneys by changing the linkers and the strategy of coordination.

## 4. Materials and Methods

### 4.1. Materials 

All chemicals in the studies were obtained from commercial sources and used without purification. [^99m^Tc]NaTcO_4_ was eluted from a ^99^Mo-^99m^Tc generator which was manufactured by Curium Netherlands B.V. (DRN 4329 Ultra-TechneKow FM., Westerduinweg, Netherlands). ESI-MS spectra were acquired by a Triple TOFTM 5600 spectrometer (AB Sciex, Singapore). The IR spectra were obtained by an IR-Affinity-1 spectrometer (Shimadzu, Kyoto, Japan). ^1^H-NMR and ^13^C-NMR spectra were acquired on a JNM-ECS spectrophotometer (JEOL, Kyoto, Japan). Radioactivity was assessed using an HRS-1000 technetium analyser (Huaruison, Beijing, China) and a Wizard 2480 γ-counter (Perkin Elmer, Singapore). HPLC analysis was carried out with an analytical column (Kromasil, 100 A - 5 μm, 250 × 4.6 mm) and a SHIMADZU system (CL-20AVP, Kyoto, Japan) equipped with an SPD-20A UV detector (λ = 254 nm) and a Bioscan flow count 3200 NaI/PMT γ-radiation scintillation detector (Eckert&Zleger Radiopharma, Washington, DC, USA). SPECT/CT imaging was recorded on a Triumph SPECT/CT scanner (TriFoil Imaging, Los Angeles, CA, USA). Female Kunming mice (18–22 g) were obtained from Beijing Vital River Laboratory Animal Technology, Beijing, China.

### 4.2. Chemistry

The synthesis of the amino derivative of 2-nitroimidazole (Compound **A**) and isocyanide-containing active esters (Compounds **B1–B5**) is shown in the Supplementary Data, and the routes to generate ligands (**L1–L5**) are shown in Scheme 1. The 2-nitroimidazole amino derivative (Compound **A**, 0.078 g, 0.5 mmol) and isocyanide-containing active esters (Compounds **B1–B5**, 0.6 mmol) were dissolved in 3 mL of methanol, and 0.2 mL of triethylamine was added. Then, the mixtures were stirred for 6–8 h at r.t. Afterwards, the solvent was evaporated under reduced pressure, and the residue was purified by column chromatography (CH_2_Cl_2_/CH_3_OH = 5:1) to obtain Ligands **L1–L5**.

Ligand **L1** (1-isocyano-N-(2-(2-nitro-1H-imidazol-1-yl)ethyl)-3,6,9,12-tetraoxapentadecan-15-amide): yellow oily liquid, yield 66%. ^1^H NMR (400 MHz, DMSO-*d*_6_) δ 7.46 (d, *J* = 1.1 Hz, 1H), 7.12 (d, *J* = 1.0 Hz, 1H), 4.47–4.31 (m, 2H), 3.64–3.39 (m, 20H), 2.20 (t, *J* = 6.4 Hz, 2H); ^13^C NMR (101 MHz, DMSO-*d*_6_) δ 171.16, 145.12, 129.14, 128.11, 70.18, 70.01, 68.48, 67.13, 49.54, 42.08, 38.60, 36.45; IR (KBr)/cm^−1^: 3269.48, 3072.74, 2912.64, 2875.99, 2152.65, 1660.78, 1537.33, 1487.18, 1361.80, 1286.58, 1163.13, 1109.12, 1028.10; HR-MS (ESI) for C_17_H_28_N_5_O_7_ [M + H]^+^: found 414.1975, calcd 414.1983.

Ligand **L2** (8-isocyano-N-(2-(2-nitro-1H-imidazol-1-yl)ethyl)octanamide): white solid, yield 87%. ^1^H NMR (400 MHz, DMSO-*d*_6_) δ 7.49 (d, *J* = 1.2 Hz, 1H), 7.11 (d, *J* = 1.1 Hz, 1H), 4.40 (dd, *J* = 6.4, 5.0 Hz, 2H), 3.44 (ddt, *J* = 9.0, 6.8, 3.6 Hz, 4H), 1.93 (dd, *J* = 9.1, 5.7 Hz, 2H), 1.52 (dt, *J* = 11.0, 6.5, 4.5, 2.3 Hz, 2H), 1.36 (p, *J* = 7.5 Hz, 2H), 1.31–1.24 (m, 2H), 1.23–1.12 (m, 4H); ^13^C NMR (101 MHz, DMSO-*d*_6_) δ 173.09, 155.87, 145.21, 129.02, 128.08, 49.65, 41.65, 38.56, 35.65, 28.91, 28.35, 26.13, 25.45; IR (KBr)/cm^−1^: 3309.09, 3078.52, 2930.00, 2856.70, 2148.79, 1653.07, 1539.26, 1489.11, 1363.73, 1273.07, 1165.05, 1082.11; HR-MS (ESI) for C_14_H_22_N_5_O_3_ [M + H]^+^: found 308.1721, calcd 308.1717.

Ligand **L3** (2-(4-(isocyanomethyl)phenyl)-N-(2-(2-nitro-1H-imidazol-1-yl)ethyl) acetamide): yellow oily liquid, yield 56%. ^1^H NMR (400 MHz, DMSO-*d*_6_) δ 7.32 (d, *J* = 1.1 Hz, 1H), 7.25 (d, *J* = 8.0 Hz, 2H), 7.18 (d, *J* = 8.2 Hz, 2H), 7.03 (d, *J* = 1.1 Hz, 1H), 4.39 (t, *J* = 5.7 Hz, 2H), 3.45 (td, *J* = 6.1, 3.0 Hz, 2H), 3.31 (s, 2H), 2.46 (s, 2H); ^13^C NMR (101 MHz, DMSO-*d*_6_) δ 170.97, 156.87, 145.15, 136.44, 132.06, 130.05, 128.86, 128.09, 127.36, 49.58, 45.23, 42.28, 40.19; IR (KBr)/cm^−1^: 3315.78, 3105.53, 2146.86, 1637.63, 1535.40, 1477.54, 1357.94, 1276.93, 1153.48, 1089.83; HR-MS (ESI) for C_15_H_16_N_5_O_3_ [M + H]^+^: found 314.1245, calcd 314.1247.

Ligand **L4** (4-(isocyanomethyl)-N-(2-(2-nitro-1H-imidazol-1-yl)ethyl)benzamide): white solid, yield 75%. ^1^H NMR (400 MHz, CD_3_OD-*d*_4_) δ 7.79–7.66 (m, 2H), 7.49–7.40 (m, 2H), 7.34 (d, *J* = 1.1 Hz, 1H), 7.06 (d, *J* = 1.1 Hz, 1H), 4.81 (d, *J* = 2.3 Hz, 2H), 4.72–4.59 (m, 2H), 3.82 (dd, *J* = 6.3, 5.0 Hz, 2H); ^13^C NMR (101 MHz, CD_3_OD-*d*_4_) δ 168.59, 156.03, 137.04, 133.77, 127.67, 127.55, 127.11, 126.65, 49.24, 44.40, 39.15; IR (KBr)/cm^−1^: 3364.00, 2945.43, 2835.48, 2150.72, 1651.14, 1548.91, 1479.49, 1356.02, 1286.58, 1253.78, 1161.20, 1028.10; HR-MS (ESI) for C_14_H_14_N_5_O_3_ [M + H]^+^: found 300.1090, calcd 300.1091.

Ligand **L5** (4-isocyano-N-(2-(2-nitro-1H-imidazol-1-yl)ethyl)benzamide): white solid, yield 63%. ^1^H NMR (400 MHz, DMSO-*d_6_*) δ 7.03–6.90 (m, 2H), 6.75–6.65 (m, 2H), 6.54 (t, *J* = 1.4 Hz, 1H), 6.25 (d, *J* = 1.3 Hz, 1H), 3.89–3.82 (m, 2H), 3.01 (td, *J* = 5.7, 1.5 Hz, 2H); ^13^C NMR (101 MHz, DMSO-*d*_6_) δ 165.69, 155.72, 135.49, 129.23, 128.18, 126.98, 49.55, 39.01; IR (KBr)/cm^−1^: 3358.21, 3275.27, 2922.28, 2852.84, 2125.65, 1653.07, 1539.26, 1489.11, 1363.73, 1282.72, 1165.13, 1097.54; HR-MS (ESI) for C_13_H_12_N_5_O_3_ [M + H]^+^: found 286.0939, calcd 286.0934.

### 4.3. Radiolabelling and Quality Control

To prepare the ^99m^Tc-complexes, the ligand was first dissolved in ethanol for obtaining a solution with a concentration of 5 mg/mL. Then, 0.1 mL of the solution (containing 0.5 mg of the ligand) was added to the reagents of a kit (including 1.0 mg of _L_-cysteine, 2.6 mg of sodium citrate, 0.1 mg of SnCl_2_·2H_2_O and 10 mg of mannitol). Freshly prepared [^99m^Tc]NaTcO_4_ (0.90 mL, 37–370 MBq) was added to the kit. It was shaken and heated at 100 °C for 20 min. The radiochemical purity was measured by TLC and HPLC. TLC was conducted using a polyamide strip (Zhejiang Taizhou Luqiao Sijia Biochemical Plastic Factory, Taizhou, China) with methanol as developing solvent. The target ^99m^Tc-complexes moved to the top (R*_f_* = 0.8–1.0), while [^99m^Tc]NaTcO_4_ and ^99m^TcO_2_·nH_2_O stayed at the origin (R*_f_* = 0–0.1). For HPLC, phase A was water, phase B was acetonitrile with a flow rate of 1 mL/min. The gradient elution profile was as follows: 0–2 min 10% B, 2–5 min 10–90% B, 5–20 min 90% B, 20–24 min 90–10% B, 24–25 min 10% B. Retention times of the ^99m^Tc-complexes: 9.54 min ([^99m^Tc]Tc-L1); 9.52 min ([^99m^Tc]Tc-L2); 9.50 min ([^99m^Tc]Tc-L3); 9.72 min ([^99m^Tc]Tc-L4); and 9.51 min ([^99m^Tc]Tc-L5).

### 4.4. Preparation of Rhenium Analogue

To validate the proposed [^99m^Tc]Tc-L1 structure, the stable complex Re-L1 was synthesised. Re-L1 was prepared as follows: 100 mg of ligand L1 was dissolved in 0.5 mL normal saline, then 1.5 mL sodium citrate buffer solution (1 M, pH = 4) and 150 μL stannous chloride solution (50 mg/mL) were added and 5 mg potassium perrhenate was added. The reaction mixture was stirred at 100 °C for 3 h. The reaction solution was separated and purified by HPLC to obtain the rhenium complex. The identification method of HPLC was the same as the method in Section 4.3. The retention time of Re-L1 is 8.94 min. The product was analysed by ^1^H NMR and MS. ^1^H NMR (600 MHz, Methanol-d4) δ 7.38 (d, *J* = 36.2 Hz, 1H), 7.11 (s, 1H), 4.54 (d, *J* = 7.2 Hz, 2H), 3.62–3.50 (m, 20H), 2.34 (d, *J* = 7.5 Hz, 2H); MS: [(C_17_H_27_N_5_O_7_)_6_ReNa_2_H]^3+^: m/z calcd 904.69, found 904.69.

### 4.5. In Vitro Stability Study 

The in vitro stability of the ^99m^Tc-complexes was determined by radiochemical purity (RCP) measurements in saline at r.t. and in mouse serum at 37 °C after 4 h. In vitro serum stability in mouse serum was determined according to a previously reported method [23]. First, 0.1 mL of the ^99m^Tc-complexes and 0.1 mL of mouse serum were mixed and incubated at 37 °C for 4 h. Afterwards, 200 µL acetonitrile was added to the centrifuge tube to precipitate the protein, and the supernatant was obtained by centrifugation. Most of the solvent was evaporated by a stream of nitrogen flow at 40 °C and diluted with 200 µL normal saline. The filtrate was filtered through a 0.22 µm microporous membrane, and the RCP of these ^99m^Tc-complexes was determined by HPLC.

### 4.6. Determination of the Partition Coefficient (Log D)

The lipophilicity of the ^99m^Tc labelled complexes was measured by determination of the partition (Log D) between *n*-octanol and phosphate buffer (PBS, pH 7.4, 0.025 mol·L^−1^), as previously reported [24,36]. First, 0.9 mL of PBS, 1.0 mL of *n*-octanol and 0.1 mL (approximately 3.7 MBq) of ^99m^Tc-complex were added to 5 mL centrifuge tubes. The mixture was centrifuged at 10,000× *g* for 5 min after vortexing for 3 min. A 0.1 mL aliquot of each phase was pipetted, and the radioactivity was measured by a γ-counter. The D value was calculated as the count of the n-octanol phase divided by that of the PBS phase. The final results were shown as Log D ± SD, n = 5.

### 4.7. In Vitro Cellular Uptake

The S180 cell line was used for in vitro cellular uptake studies. The cells were incubated separately in hypoxic and aerobic conditions. The hypoxic group was incubated in an atmosphere of 95% nitrogen and 5% carbon dioxide (oxygen concentration < 10 ppm, measured by a dissolved oxygen meter), while the aerobic group was incubated in an atmosphere of 95% air and 5% carbon dioxide. The S180 cells were suspended in 20 mL of Dulbecco’s modified Eagle medium (DMEM) with 10% (*v*/*v*) fetal bovine serum (FBS) to form a solution with a concentration of 2 × 10^6^/mL, and they were incubated at 37 °C. Then, 0.2 mL of ^99m^Tc-labelled complex (3.7 MBq) was added into the glass vials, and the chemical concentration of ligands was 6–9 nM. After incubation for 0.5, 1, 2 and 4 h, 1 mL of cell suspension was removed with a pipette and centrifuged at 3000× *g* for 5 min. Then, the radioactivity of 0.9 mL of the supernatant was taken for counting (C_out_), and the radioactivity of the residual sample containing 0.1 mL of the supernatant and cells was also taken for counting (C_in_). Samples were taken and measured five times at each time point. Cell uptake was expressed according to the following formula: uptake (%) = (C_in_ − C_out_/9)/(C_in_ + C_out_) × 100%. The results are presented as the mean value ± standard deviation.

### 4.8. Biodistribution Studies

For biodistribution studies, female Kunming mice bearing S180 tumours weighing approximately 18 to 22 g were used. Approximately 1 × 10^6^ S180 cells were subcutaneously injected into the left anterior axilla of mice. About one week later, the diameter of the tumour was 5–8 mm, and the mice were used for further experiments.

Into each mouse, 0.1 mL of ^99m^Tc-complexes (3.7 MBq·mL^−1^) was injected via a tail vein. They were sacrificed after anaesthesia at 0.5 h or 2 h post-injection with five mice in each group. The tumour, blood, muscle, liver, kidneys, heart, lung, spleen, stomach, bone, small intestine, large intestine and other tissues of interest were collected, weighed and measured by a γ-counter. The final results are expressed as the percentage uptake of the injected dose per gram of tissue ± standard deviation (%ID/g ± SD).

### 4.9. SPECT/CT Imaging Studies

SPECT/CT imaging studies were performed using a Triumph SPECT/CT scanner, reconstructed with HiSPECT software (Bioscan, Washington, DC, USA) and processed with Vivoquant 2.5 software (Invicro, Needham, MA, USA). The mice used for the SPECT/CT imaging studies were anaesthetised and maintained under anaesthesia during the imaging procedure. The SPECT/CT imaging was performed with female Kunming mice bearing S180 tumours at 2 h post-injection of [^99m^Tc]Tc-L1 (0.1 mL, 14.8 MBq).

### 4.10. In Vivo Metabolic Studies 

[^99m^Tc]Tc-L1 (0.2 mL, 111 MBq) was injected into Kunming mice bearing S180 tumours via a tail vein. After 2 h, the mice were sacrificed, then urine, blood and tumour were collected. The urine was passed through a 0.22 µm microporous membrane and analysed by HPLC. The blood was centrifuged at 10,000× *g* for 3 min, then 100 μL of the supernatant was mixed with 200 μL of acetonitrile. After centrifugation, the protein was precipitated and removed, and the supernatant was collected and passed through a 0.22 µm microporous membrane, then it was concentrated and analysed by HPLC. The tumours were homogenised by a TH-02 tissue-tearor (Omini, Kennesaw, GA, USA) with 200 μL saline, then 400 μL of acetonitrile was added into the centrifuge tube and the mixture was centrifuged to precipitate the protein, and the supernatant was collected after centrifugation. Then, the supernatant was passed through a 0.22 µm microporous membrane, concentrated by rotary evaporator and analysed by HPLC. The method of HPLC was the same as the method in Section 4.3.

## 5. Conclusions

In this study, five isonitrile hypoxia ligands with different linkers were designed, synthesised and radiolabelled with ^99m^Tc in a single step over 20 min to obtain five stable ^99m^Tc-complexes. The study of corresponding rhenium analogues proved the structures of these ^99m^Tc-complexes would be a monovalent cation with a technetium (I) core that is surrounded by six ligands. All of the complexes had good hypoxia selectivity and certain uptake in the tumours. Among them, [^99m^Tc]Tc-L1 had the highest hydrophilicity and the highest tumour-to-muscle and tumour-to-blood ratios. And it also stayed intact and remained stable in tumour, blood and urine at 2 h post-injection. The SPECT/CT imaging results showed that [^99m^Tc]Tc-L1 was obviously taken up in the tumour site, but there was still the problem of high uptake in the liver and abdomen. Further exploration of structural modifications of the ligand and the linker is necessary.

## Figures and Tables

**Table 1:** Log D ± SD values of the ^99m^Tc-complexes.

Complexes	Log D ± SD
[^99m^Tc]Tc-L1	−2.70 ± 0.22
[^99m^Tc]Tc-L2	−0.18 ± 0.05
[^99m^Tc]Tc-L3	−0.06 ± 0.02
[^99m^Tc]Tc-L4	0.10 ± 0.02
[^99m^Tc]Tc-L5	0.24 ± 0.08

**Table 2:** Biodistribution of the ^99m^Tc tracers in female Kunming mice bearing S180 tumours at 0.5 h and 2 h post-injection (%ID/g ± SD, n = 5).

	[^99m^Tc]Tc-L1		[^99m^Tc]Tc-L2		[^99m^Tc]Tc-L3		[^99m^Tc]Tc-L4		[^99m^Tc]Tc-L5	
	0.5 h	2 h	0.5 h	2 h	0.5 h	2 h	0.5 h	2 h	0.5 h	2 h
Heart	0.43 ± 0.12	0.12 ± 0.03	1.19 ± 0.20	0.82 ± 0.10	0.35 ± 0.02	0.26 ± 0.11	0.88 ± 0.09	0.56 ± 0.05	0.59 ± 0.20	0.32 ± 0.05
Liver	3.71 ± 0.47	3.49 ± 0.45	35.36 ± 5.42	33.31 ± 3.75	11.26 ± 0.54	11.21 ± 1.07	19.62 ± 3.43	17.16 ± 2.36	45.08 ± 8.90	39.67 ± 8.11
Lung	0.86 ± 0.02	0.24 ± 0.02	2.78 ± 0.52	4.32 ± 1.90	1.33 ± 0.31	0.88 ± 0.19	2.76 ± 0.14	1.27 ± 0.13	2.76 ± 1.19	1.71 ± 1.44
Kidneys	10.08 ± 1.14	8.93 ± 0.80	20.10 ± 1.97	24.70 ± 2.77	8.55 ± 0.67	4.87 ± 0.77	11.85 ± 2.18	10.15 ± 1.54	1.39 ± 0.17	0.96 ± 0.09
Spleen	0.84 ± 0.07	0.65 ± 0.03	2.83 ± 0.81	3.09 ± 0.51	2.11 ± 0.34	1.78 ± 0.41	2.53 ± 0.25	1.79 ± 0.16	36.16 ± 3.80	27.51 ± 7.68
Stomach	0.50 ± 0.08	0.41 ± 0.36	1.21 ± 0.46	3.16 ± 2.60	0.45 ± 0.28	0.22 ± 0.07	0.55 ± 0.12	0.63 ± 0.10	0.66 ± 0.23	0.30 ± 0.13
Bone	0.77 ± 0.09	0.47 ± 0.04	2.08 ± 0.32	1.46 ± 0.24	0.89 ± 0.09	0.73 ± 0.19	1.25 ± 0.14	1.11 ± 0.13	0.70 ± 0.12	0.58 ± 0.17
Muscle	0.32 ± 0.08	0.10 ± 0.04	0.99 ± 0.58	0.46 ± 0.13	0.22 ± 0.05	0.16 ± 0.07	0.31 ± 0.04	0.23 ± 0.03	0.30 ± 0.06	0.18 ± 0.04
Small Intestine	1.34 ± 0.62	0.45 ± 0.15	3.74 ± 1.15	2.93 ± 1.34	1.70 ± 0.30	1.05 ± 0.31	2.04 ± 0.23	2.06 ± 0.21	0.64 ± 0.15	0.21 ± 0.04
Large Intestine	0.71 ± 0.12	2.49 ± 0.20	0.83 ± 0.30	7.36 ± 2.09	0.23 ± 0.04	3.11 ± 0.85	0.31 ± 0.11	2.40 ± 0.25	0.29 ± 0.06	0.72 ± 0.14
Tumour	1.11 ± 0.13	0.47 ± 0.10	1.22 ± 0.22	1.19 ± 0.24	0.40 ± 0.10	0.31 ± 0.09	1.05 ± 0.19	0.97 ± 0.13	0.33 ± 0.09	0.31 ± 0.09
Blood	0.80 ± 0.06	0.13 ± 0.00	2.23 ± 0.44	1.38 ± 0.06	0.56 ± 0.04	0.23 ± 0.04	1.22 ± 0.01	0.85 ± 0.14	0.38 ± 0.08	0.17 ± 0.02
Thyroid (%ID)	0.05 ± 0.02	0.01 ± 0.00	0.04 ± 0.01	0.03 ± 0.02	0.04 ± 0.04	0.03 ± 0.02	0.06 ± 0.01	0.03 ± 0.01	0.05 ± 0.02	0.03 ± 0.01
Tumour/Muscle	4.10 ± 0.63	4.68 ± 0.44	1.24 ± 0.50	2.44 ± 0.05	1.87 ± 0.51	2.32 ± 0.62	2.84 ± 0.41	4.15 ± 0.54	1.32 ± 0.17	1.72 ± 0.30
Tumour/Blood	1.38 ± 0.10	3.81 ± 0.46	0.55 ± 0.05	0.87 ± 0.18	0.71 ± 0.12	1.42 ± 0.12	0.64 ± 0.05	1.16 ± 0.22	1.00 ± 0.17	1.79 ± 0.46

## Data Availability

Data is contained within the article.

## Supplementary Materials

The following are available online at: https://www.mdpi.com/article/10.3390/ph16091276/s1, Scheme S1. The synthesis of Compound **A**; Scheme S2. The synthesis of Compound **B1**; Scheme S3. The synthesis of Compound **B2**; Scheme S4. The synthesis of Compound **B3**; Scheme S5. The synthesis of Compound **B4**; Scheme S6. The synthesis of Compound **B5**; Figure S1. HR-MS chromatogram spectrum of **L1**; Figure S2. HR-MS chromatogram spectrum of **L2**; Figure S3. HR-MS chromatogram spectrum of **L3**; Figure S4. HR-MS chromatogram spectrum of **L4**; Figure S5. HR-MS chromatogram spectrum of **L5**; Figure S6. ^1^H NMR spectrum of **L1**; Figure S7. ^13^C NMR spectrum of **L1**; Figure S8. ^1^H NMR spectrum of **L2**; Figure S9. ^13^C NMR spectrum of **L2**; Figure S10. ^1^H NMR spectrum of **L3**; Figure S11. ^13^C NMR spectrum of **L3**; Figure S12. ^1^H NMR spectrum of **L4**; Figure S13. ^13^C NMR spectrum of **L4**; Figure S14. ^1^H NMR spectrum of **L5**; Figure S15. ^13^C NMR spectrum of **L5**; Figure S16. ^1^H NMR spectrum of Re-L1; Figure S17. MS chromatogram spectrum of Re-L1; Table S1. P-values of cellular uptake at different time points; Table S2. Biodistribution of [^99m^Tc]Tc-L1 in female Kunming mice bearing S180 tumours at 0.5 h, 2 h and 6 h postinjection (%ID/g ± SD, n = 5); Figure S18. Maximum intensity projection of SPECT images of [^99m^Tc]Tc-L1 at 0.5 h, 1 h and 2 h post-injection.
