# Supplementary material for: Synthesis and Evaluation of 99mTc-Labelled 2-Nitroimidazole Derivatives with Different Linkers for Tumour Hypoxia Imaging
Source: Pharmaceuticals (Basel). 2023 Sep 9;16(9):1276. doi: 10.3390/ph16091276 (PMC10537343; doi:10.3390/ph16091276)
Supplement: Supplementary file 1 [file pharmaceuticals-16-01276-s001.zip › pharmaceuticals-2560711-supplementary.pdf]

Supplementary Data

Synthesis and Evaluation of ^{99m}Tc-Labelled 2-Nitroimidazole Derivatives with Different Linkers for Imaging of Tumour Hypoxia

Qing Ruan[1,2,#], Yitong Liu[2,#], Lihao Liao[2], Jinyu Hao[2], Yuhao Jiang[2], Jianyong Jiang[1,*], Junbo Zhang[2,*]

[1] Key Laboratory of Beam Technology of the Ministry of Education, College of Nuclear Science and Technology, Beijing Normal University, Beijing, 100875, P. R. China

[2] Key Laboratory of Radiopharmaceuticals of the Ministry of Education, NMPA Key Laboratory for Research and Evaluation of Radiopharmaceuticals (National Medical Products Administration), College of Chemistry, Beijing Normal University, Beijing, 100875, P. R. China

[#]**First authors:** Qing Ruan, Yitong Liu contributed equally to this work.

[*]**Corresponding author:**

Jianyong Jiang, Email: jianyong@bnu.edu.cn. Key Laboratory of Beam Technology of the Ministry of Education, College of Nuclear Science and Technology, Beijing Normal University, Beijing, 100875, P. R. China

Junbo Zhang, Email: zhjunbo@bnu.edu.cn. Key Laboratory of Radiopharmaceuticals of the Ministry of Education, NMPA Key Laboratory for Research and Evaluation of Radiopharmaceuticals (National Medical Products Administration), College of Chemistry, Beijing Normal University, Beijing, 100875, P. R. China

Content

Synthesis of Compound A. S3

Synthesis of Compound B1. .... S3

Synthesis of Compound B2. .... S4

Synthesis of Compound B3. .... S5

Synthesis of Compound B4. .... S6

Synthesis of Compound B5 .... S7

Figure S1. HR-MS spectrum of L1. .... S8

Figure S2. HR-MS spectrum of L2 .... S9

Figure S3. HR-MS spectrum of L3 .... S9

Figure S4. HR-MS spectrum of L4 .... S10

Figure S5. HR-MS spectrum of L5 .... S10

Figure S6. and S7. ^{1}H NMR and ^{13}C NMR spectra of L1. .... S11

Figure S8. and S9. ^{1}H NMR and ^{13}C NMR spectra of L2. .... S12

Figure S10. and S11. ^{1}H NMR and ^{13}C NMR spectra of L3. .... S13

Figure S12. and S13. ^{1}H NMR and ^{13}C NMR spectra of L4. .... S14

Figure S14. and S15. ^{1}H NMR and ^{13}C NMR spectra of L5. .... S15

Figure S16. ^{1}H NMR and Figure S17. MS spectra of Re-L1. .... S16

Table S1. P-values of cellular uptake. S17

Table S2. Biodistribution of [^{99m}Tc]Tc-L1 at 0.5, 2 and 6 h post-injection. S17

Figure S18. SPECT images of [^{99m}Tc]Tc-L1 at 0.5 h, 1 h and 2 h post-injection S18

Synthesis of Compound A

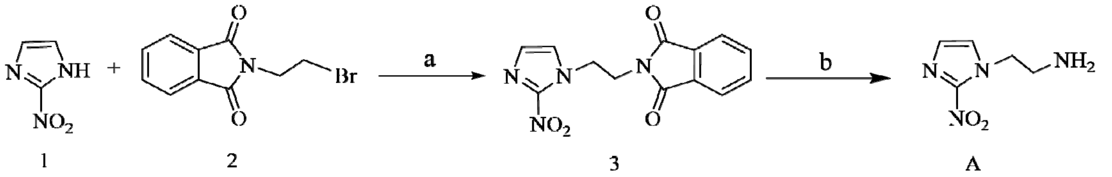

Scheme S1. The synthesis of Compound A. Reagents and conditions: (a) DMF, K_2CO_3, reflux, 3 h; (b) EtOH, hydrazine hydrate, reflux, 4 h.

Compound 3. Compound 1 (2-nitro-1*H*-imidazole, 1.13 g, 10 mmol) and Compound 2 (2-(2-bromoethyl)isoindoline-1,3-dione, 3.05 g, 12 mmol) were added into DMF (50 mL). After an addition of K_2CO_3 (0.98 g, 12 mmol), the mixture was refluxed for 3 h at 110 °C. The solvent was cooled to room temperature before the solid was filtered from the solution. Then, the solid was washed with water and dried to give crude product (Compound 3, 2-(2-(2-nitro-1*H*-imidazol-1-yl)ethyl) isoindoline-1,3-dione, 2.56 g, 89 %). ^{1}H NMR (400 MHz, DMSO-d_6) δ 7.79 (s, 4H), 7.56 (d, J = 1.1 Hz, 1H), 7.02 (d, J = 1.1 Hz, 1H), 4.67-4.46 (m, 2H), 4.11-3.87 (m, 2H).

Compound A. Compound 3 (1.43 g, 5 mmol) and hydrazine hydrate (0.50 g, 10 mmol) were dissolved in ethanol (50 mL). Then, the mixture was refluxed for 4 h. After cooling to room temperature, the mixture was stored in a refrigerator at 0 °C for 12 h. After the insoluble substance was removed by filtration, the solvent was removed under a reduced pressure and the residue was purified by silica gel column chromatography (CH_2Cl_2/CH_3OH) to give Compound 1 (2-(2-nitro-1*H*-imidazol-1-yl)ethan-1-amine, 0.61g, 77%). ^{1}H-NMR (600 MHz, D_2O) δ 8.05 (s, 1H), 7.60 (s, 1H), 4.06 (t, J = 6.0 Hz, 2H), 2.96 (t, J = 5.9 Hz, 2H); MS (m/z): $[M + H]^+$ 157.01.

Synthesis of Compound B1

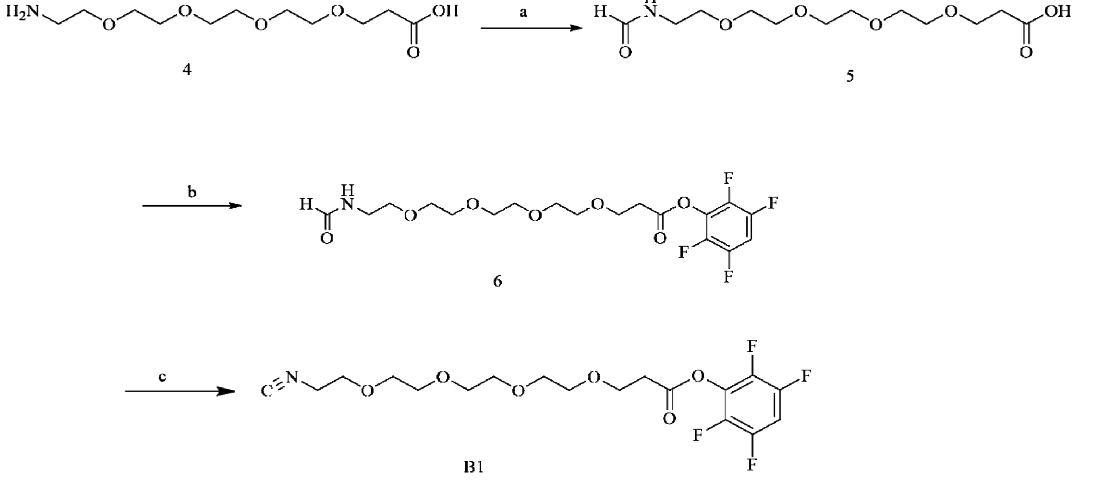

Scheme S2. The synthesis of Compound B1. Reagents and conditions: (a) Formic acid, DMF, 140 °C, 5 h; (b) TFP, EDCI, DCM, r.t., overnight; (c) Burgess reagent, DCM, r.t., 4 h.

Compound 5. Compound 4 (1.86 g, 7.00 mmol) and formic acid (0.48 g, 10.50 mmol) were added to a 25 mL flask with DMF (10 mL) and refluxed at 140 °C for 5 h. The solvent was removed under vacuum and the oily residue was purified by silica gel column chromatography using petroleum dichloromethane/methanol (10:1, V/V) as the mobile phase to afford a brown oily product (Compound 5, 1.69 g, 82%). ^{1}H NMR (400 MHz, D_2O) δ 8.07 (s, 1H), 3.80 (t, J = 6.0 Hz, 3H), 3.69 (s, 5H), 3.65 (dd, J = 8.6, 3.1 Hz, 4H), 3.44 (t, J = 5.3 Hz, 3H), 3.35 (t, J = 0.5 Hz, 1H), 2.67 (t, J = 6.0 Hz, 3H), 1.17 (td, J = 7.1, 0.4 Hz, 1H).

Compound 6. Compound 5 (1.686 g, 5.71 mmol) and 2,3,5,6-tetraflfluorophenol (TFP, 0.95 g, 5.71 mmol) were mixed in DCM (25 mL) solution. Then, *N*-(3-dimethylaminopropyl)-*N'*-ethylcarbodiamide hydrochloride (EDCI, 1.31 g, 6.80 mmol) was added to the mixture in an ice bath and stirred at room temperature (r.t.) overnight. The reaction solvent was removed under vacuum and the oily residue was purified by silica gel column chromatography using petroleum ether/ethyl acetate (5:1, V/V) as the mobile phase to obtain a brown oily product (Compound 6, 1.67 g, 66%). ^{1}H-NMR (400 MHz, CD_3OD) δ 8.07 (s, 1H), 7.50 – 7.38 (m, 1H), 3.89 (td, J = 6.0, 1.8 Hz, 2H), 3.68 – 3.64 (m, 12H), 3.59 – 3.55 (m, 2H), 3.41 (t, J = 5.2 Hz, 2H), 3.00 (td, J = 6.0, 1.8 Hz, 2H).

Compound B1. Compound 6 (1.66 g, 3.82 mmol) and Burgess reagent (1.07 g, 4.5 mmol) were mixed in DCM (20 mL) and reacted at r.t. for 4 h. The solvent was removed under vacuum and the oily residue was purified by silica gel column chromatography using petroleum ether/ethyl acetate (5:1, V/V) as the mobile phase to afford Compound B1 as a brown oily product (1.24 g, 78%). ^{1}H-NMR (400 MHz, CD_3OD) δ 7.45 (m, 1H), 3.90 (td, J = 6.0, 1.5 Hz, 2H), 3.70 – 3.63 (m, 16H), 3.00 (td, J = 6.0, 1.4 Hz, 2H).

Synthesis of Compound B2

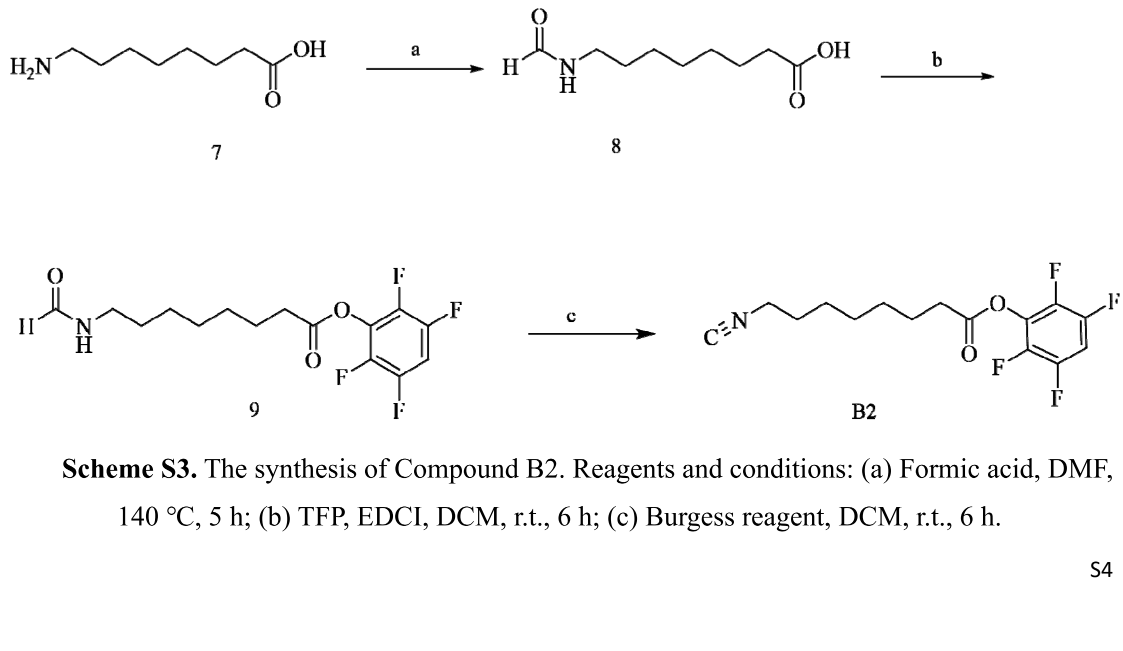

Scheme S3. The synthesis of Compound B2. Reagents and conditions: (a) Formic acid, DMF, 140 °C, 5 h; (b) TFP, EDCI, DCM, r.t., 6 h; (c) Burgess reagent, DCM, r.t., 6 h.

Compound 8. Compound 7 (3.00 g, 18.85 mmol) and formic acid (1.60 g, 28.28 mmol) were added to a 25 mL flask with DMF (10 mL) and refluxed at 140 °C for 5 h. After the reaction, it was put in the refrigerator overnight to be crystallised out. The filter residue was collected by filtration and then washed with ethyl acetate (50 mL × 3) to obtain the product as a white solid (Compound 8, 2.53 g, 72%). ^{1}H NMR (600 MHz, DMSO-d_6) δ 12.01 (s, 1H), 8.07-7.80 (m, 2H), 3.04 (q, J = 7.8 Hz, 2H), 2.15 (t, J = 7.4 Hz, 2H), 1.50-1.44 (m, 2H), 1.42-1.34 (m, 2H), 1.31 (s, 6H).

Compound 9. Compound 8 (2.52 g, 13.58 mmol) and TFP (2.05 g, 12.34 mmol) were mixed in DCM (25 mL) solution. Then, EDCI (2.84 g, 14.81 mmol) was added to the mixture in an ice bath and stirred at r.t. for 6 h. The reaction solution was washed with sodium bicarbonate solution and sodium chloride solution in sequence, then the organic phase was collected and the solvent was removed by vacuum rotary evaporation to obtain the product as a white solid (Compound 9, 3.17 g, 66%). ^{1}H NMR (600 MHz, $CDCl_3$) δ 8.15 (s, 1H), 6.97 (tt, J = 9.9, 7.0 Hz, 1H), 5.52 (s, 1H), 3.30 (dq, J = 50.7, 6.8 Hz, 2H), 2.65 (td, J = 7.4, 3.0 Hz, 2H), 1.77 (dt, J = 15.0, 7.4 Hz, 2H), 1.55-1.52 (m, 2H), 1.43-1.41 (m, 2H), 1.40-1.36 (m, 4H).

Compound B2. Compound 9 (3.16 g, 9.42 mmol) and Burgess reagent (2.47 g, 10.37 mmol) were mixed in DCM (25 mL) and reacted at r.t. for 6 h. The solvent was removed under vacuum and the oily residue was purified by silica gel column chromatography using petroleum ether/ethyl acetate (10:1, V/V) as the mobile phase to afford Compound B2 as a brown solid (2.24 g, 76%). ^{1}H NMR (400 MHz, $CDCl_3$) δ 6.98 (tt, J = 9.9, 7.1 Hz, 1H), 3.41-3.39 (m, 2H), 2.67 (t, J = 7.3 Hz, 2H), 1.78 (dt, J = 14.9, 7.3 Hz, 2H), 1.74-1.66 (m, 2H), 1.52-1.36 (m, 6H).

Synthesis of Compound B3

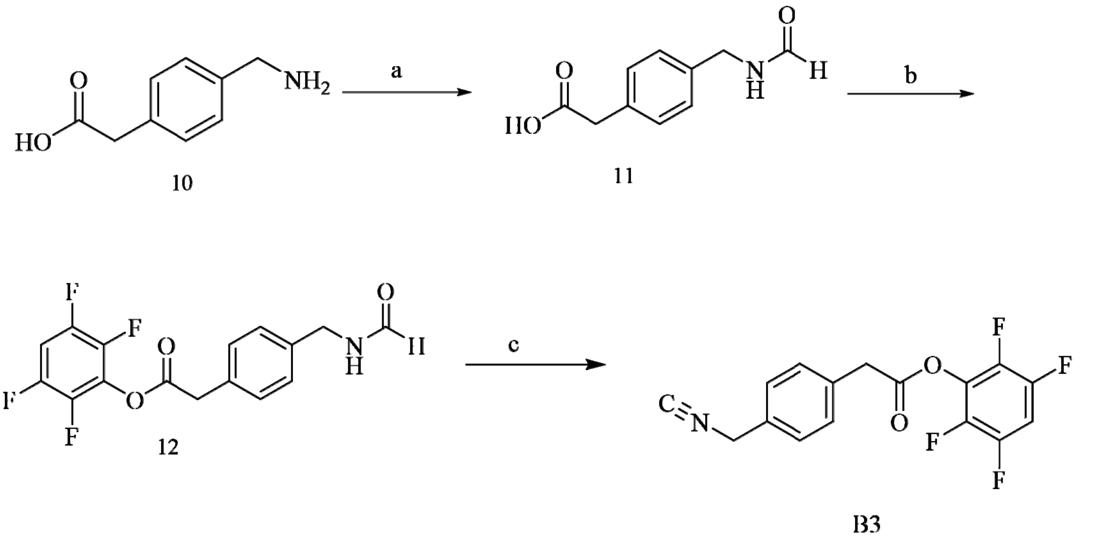

Scheme S4. The synthesis of Compound B3. Reagents and conditions: (a) Formic acid, acetic anhydride, r.t., 3 h; (b) TFP, EDCI, DCM, 6 h; (c) Burgess reagent, DCM, r.t., 6 h.

Compound 11. Compound 10 (2.97 g, 18.00 mmol) and formic acid (10 mL) were added to a 25 mL flask and stirred at 60 °C for 10 min. After the reaction solution was cooled to room temperature, 4 mL acetic anhydride was added and the reaction was carried out for 3 h at room temperature, and then the white solid was crystallised out. The filter residue was collected by filtration and then washed with ethyl acetate (50 mL × 3) to obtain the product as a white solid (Compound 11, 2.65 g, 40%). ^{1}H NMR (400 MHz, DMSO-d_6) δ 8.49 (s, 1H), 8.13 (s, 1H), 7.21 (s, 4H), 4.28 (d, J = 6.1 Hz, 2H), 3.55 (s, 2H).

Compound 12. Compound 11 (2.50 g, 12.95 mmol) and TFP (1.95 g, 11.77 mmol) were mixed in DCM (15 mL) solution. Then, EDCI (2.70 g, 14.12 mmol) was added to the mixture in an ice bath and stirred at r.t. for 6 h. The reaction solution was washed with sodium bicarbonate solution and sodium chloride solution in sequence, then the organic phase was collected and the solvent was removed by vacuum rotary evaporation to obtain the product as a white solid (Compound 12, 3.17 g, 86%). ^{1}H NMR (400 MHz, DMSO-d_6) δ 8.53 (s, 1H), 8.15- 8.13 (m, 1H), 7.95 (tt, J = 11.0, 7.5 Hz, 1H), 7.36 (d, J = 8.2 Hz, 2H), 7.29 (d, J = 8.3 Hz, 2H), 4.32 (d, J = 6.0 Hz, 2H), 4.19 (s, 2H).

Compound B3. Compound 12 (3.09 g, 9.05 mmol) and Burgess reagent (2.37 g, 9.96 mmol) were mixed in DCM (25 mL) and reacted at r.t. for 6 h. The solvent was removed under vacuum and the oily residue was purified by silica gel column chromatography using petroleum ether/ethyl acetate (10:1, V/V) as the mobile phase to afford Compound B3 as a light yellow oily product (2.24 g, 75%). ^{1}H NMR (600 MHz, $CDCl_3$) δ 7.44 (d, J = 8.2 Hz, 2H), 7.39 (d, J = 8.2 Hz, 2H), 7.02 (tt, J = 9.8, 7.0 Hz, 1H), 4.67 (s, 2H), 4.01 (s, 2H).

Synthesis of Compound B4

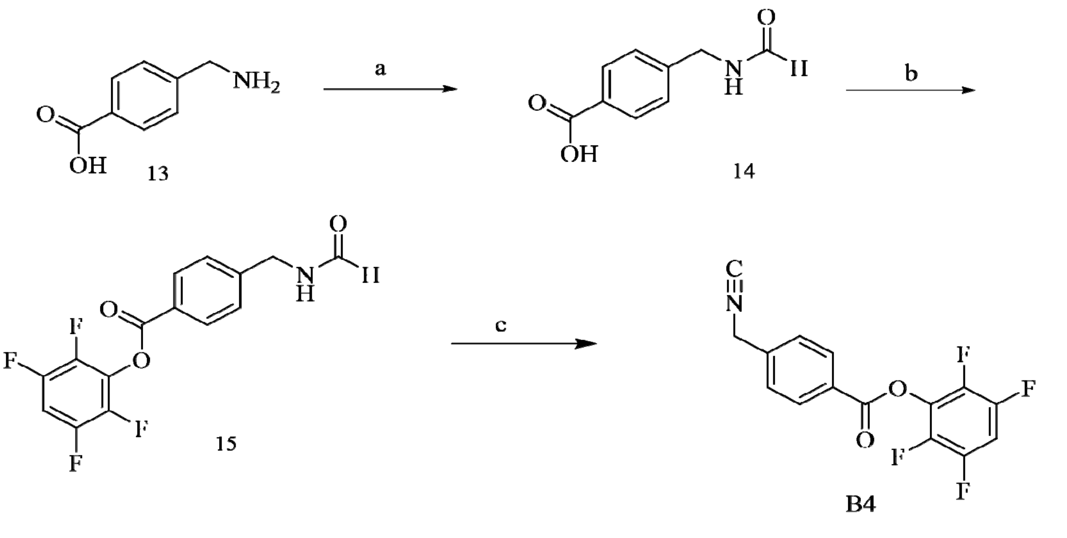

Scheme S5. The synthesis of Compound B4. Reagents and conditions: (a) Formic acid, DMF, 140 °C, 3 h; (b) TFP, EDCI, DCM, r.t., 6 h; (c) Burgess reagent, DCM, r.t., 5 h.

Compound 14. Compound 13 (1.00 g，6.62 mmol) and formic acid (0.46 g，9.93 mmol) were added to a 25 mL flask with DMF (5 mL) and refluxed at 140 °C for 3 h. After the reaction, it was kept at room temperature to be crystallised out. The filter residue was collected by filtration and then washed with ethyl acetate (50 mL × 3) to obtain the product as a white solid (Compound 14, 0.75 g，63%). ^{1}H NMR (400 MHz, DMSO-d_6) δ 8.54 (t, J = 6.4 Hz, 1H), 8.13 (d, J = 1.6 Hz, 1H), 7.92-7.75 (m, 2H), 7.41-7.24 (m, 2H), 4.33 (d, J = 6.2 Hz, 2H).

Compound 15. Compound 14 (0.68 g，3.79 mmol) and TFP (0.75 g，3.45 mmol) were mixed in DCM (5 mL) solution. Then, EDCI (0.86 g， 4.50 mmol) was added to the mixture in an ice bath and stirred at r.t. for 6 h. The reaction solution was washed with sodium bicarbonate solution and sodium chloride solution in sequence, then the organic phase was collected and the solvent was removed by vacuum rotary evaporation to obtain the product as a white solid (Compound 15, 0.92 g， 81%). ^{1}H NMR (400 MHz, $CDCl_3$) δ 8.23-8.04 (m, 2H), 7.55-7.38 (m, 2H), 7.03 (tt, J = 9.9, 7.0 Hz, 1H), 4.62-4.45 (m, 2H).

Compound B4. Compound 15 (0.90 g，2.75 mmol) and Burgess reagent (0.72 g，3.03 mmol) were mixed in DCM (5 mL) and reacted at r.t. for 5 h. The solvent was removed under vacuum and the oily residue was purified by silica gel column chromatography using petroleum ether/ethyl acetate (10:1, V/V) as the mobile phase to afford Compound B4 as a white solid (0.64 g，75%). ^{1}H NMR (400 MHz, $CDCl_3$) δ 8.29-8.21 (m, 2H), 7.54 (dq, J = 7.5, 0.8 Hz, 2H), 7.05 (tt, J = 9.9, 7.0 Hz, 1H), 4.77 (s, 2H).

Synthesis of Compound B5

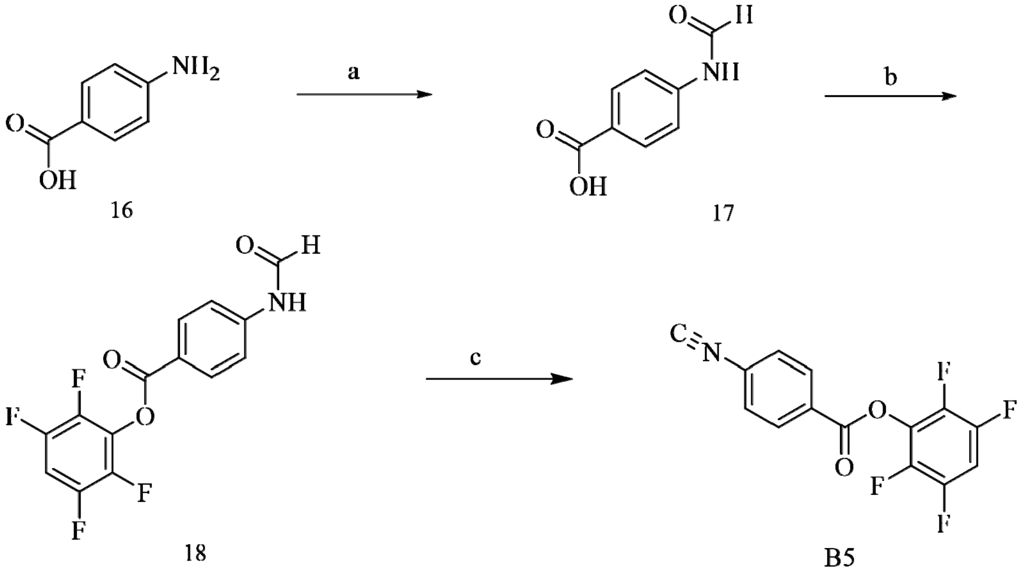

Scheme S6. The synthesis of Compound B5. Reagents and conditions: (a) Formic acid, acetic anhydride, r.t., 3 h; (b) TFP, EDCI, DCM, r.t., 6 h; (c) Burgess reagent, DCM, r.t., 6 h.

Compound 17. Compound 16 (5.00 g，36.48 mmol) and formic acid (10 mL) were added to a 25 mL flask and stirred at 60 °C for 10 min. After the reaction solution was cooled to room temperature, 4 mL acetic anhydride was added and the reaction was carried out for 3 h at room temperature, and then the white solid was crystallised out. The filter residue was collected by filtration and then washed with ethyl acetate (50 mL × 3) to obtain the product as a white solid (Compound 17, 3.03 g，50%). ^{1}H NMR (400 MHz, DMSO-d_6) δ 12.73 (s, 1H), 10.45 (s, 1H), 8.30 (d, J = 1.8 Hz, 1H), 7.91-7.86 (m, 2H), 7.64 (dt, J = 8.8, 2.4 Hz, 2H).

Compound 18. Compound 17 (2.95 g，17.87 mmol) and TFP (2.69 g，16.25 mmol) were mixed in DCM (15 mL) solution. Then, EDCI (3.74 g，19.50 mmol) was added to the mixture in an ice bath and stirred at r.t. for 6 h. The reaction solution was washed with sodium bicarbonate solution and sodium chloride solution in sequence, then the organic phase was collected and the solvent was removed by vacuum rotary evaporation to obtain the product as a white solid (Compound 18, 4.53 g，89%). ^{1}H NMR (400 MHz, DMSO-d_6) δ 10.74 (s, 1H), 8.39 (d, J = 1.6 Hz, 1H), 8.16-8.09 (m, 2H), 7.98 (tt, J = 11.0, 7.5 Hz, 1H), 7.83 (d, J = 8.7 Hz, 2H).

Compound B5. Compound 18 (4.41 g，14.09 mmol) and Burgess reagent (4.03 g， 16.91 mmol) were mixed in DCM (25 mL) and reacted at r.t. for 6 h. The solvent was removed under vacuum and the oily residue was purified by silica gel column chromatography using petroleum ether/ethyl acetate (10:1, V/V) as the mobile phase to afford Compound B5 as a light yellow oily product (3.31 g， 79%). ^{1}H NMR (400 MHz, $CDCl_3$) δ 8.29-8.16 (m, 2H), 7.51 (dd, J = 34.3, 20.9 Hz, 2H), 7.08-7.04 (m, 1H).

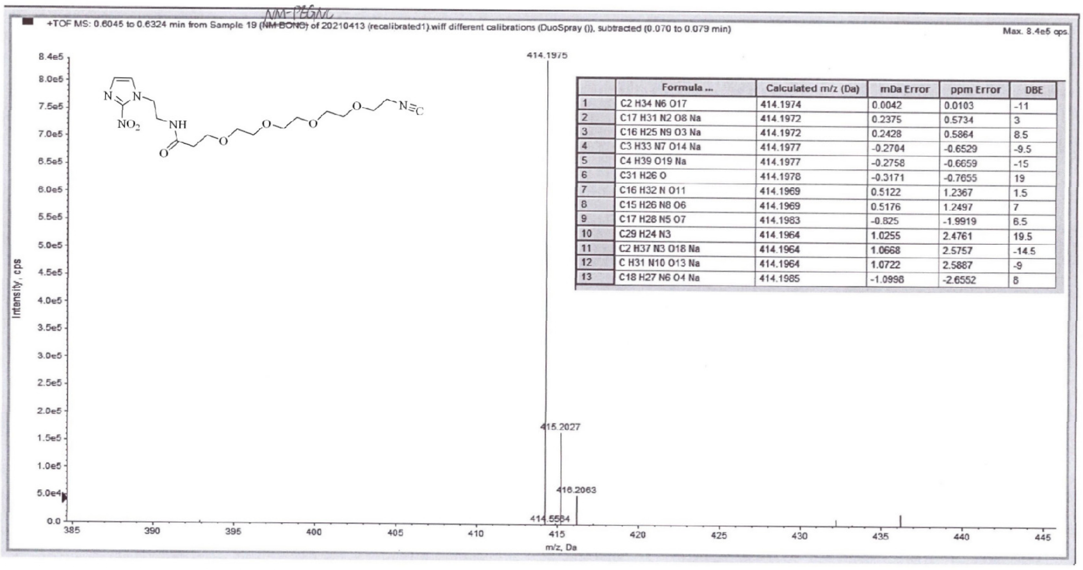

Figure S1. HR-MS chromatogram spectrum of L1.

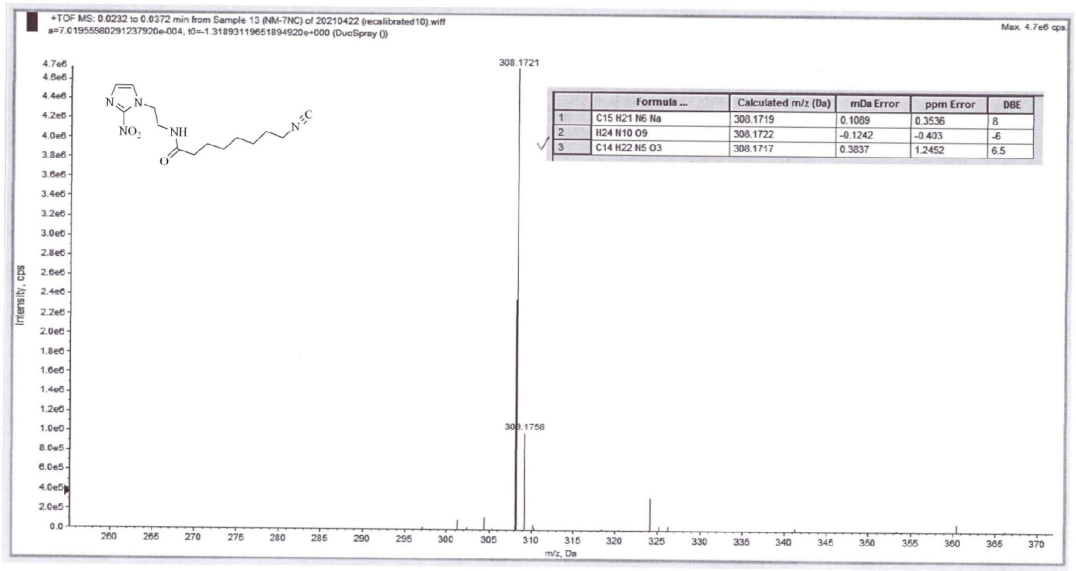

Figure S2. HR-MS chromatogram spectrum of L2.

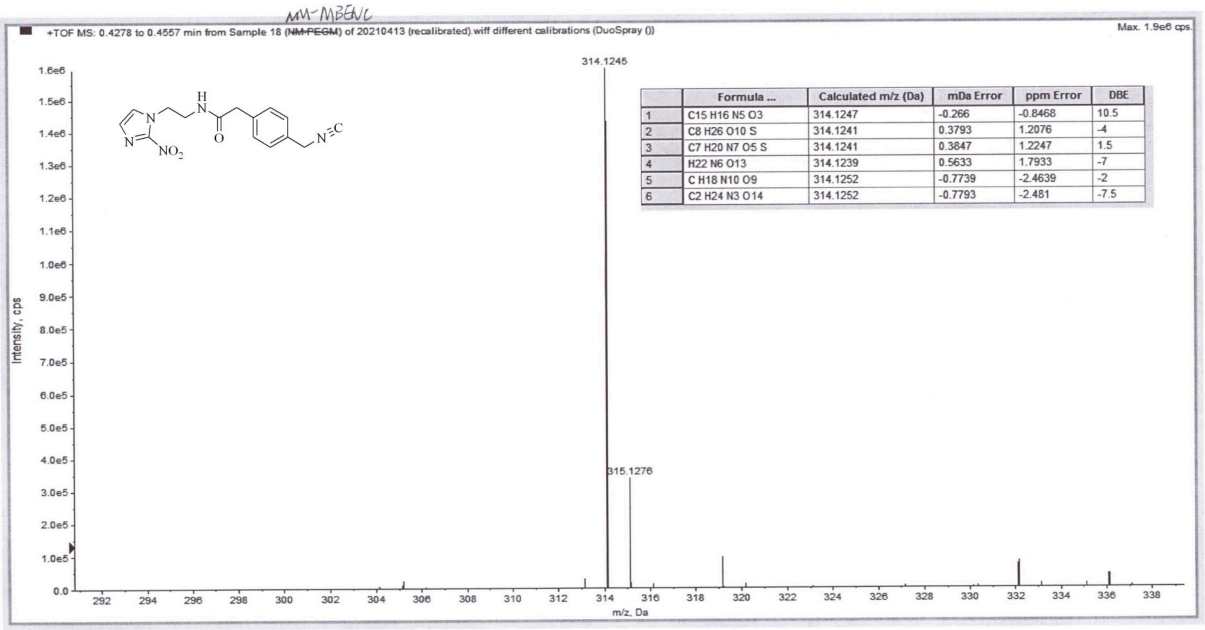

Figure S3. HR-MS chromatogram spectrum of L3.

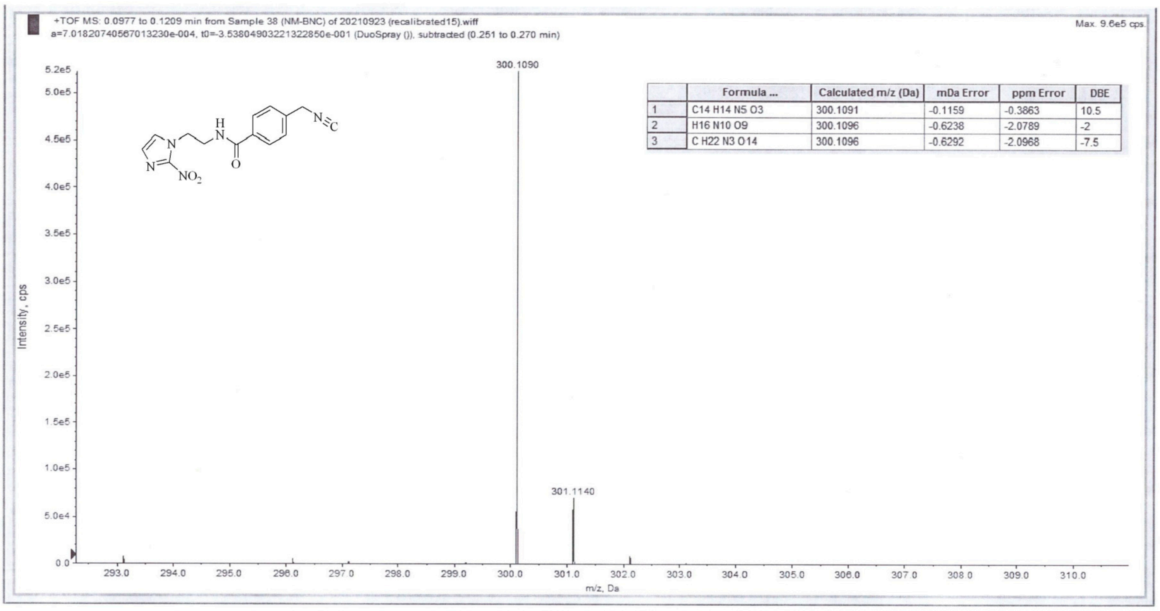

	Formula ...	Calculated m/z (Da)	mDa Error	ppm Error	DBE
1	C14 H14 N5 O3	300.1091	-0.1159	-0.3863	10.5
2	H16 N10 O9	300.1096	-0.6238	-2.0789	-2
3	C H22 N3 O14	300.1096	-0.6292	-2.0968	-7.5

Figure S4. HR-MS chromatogram spectrum of L4.

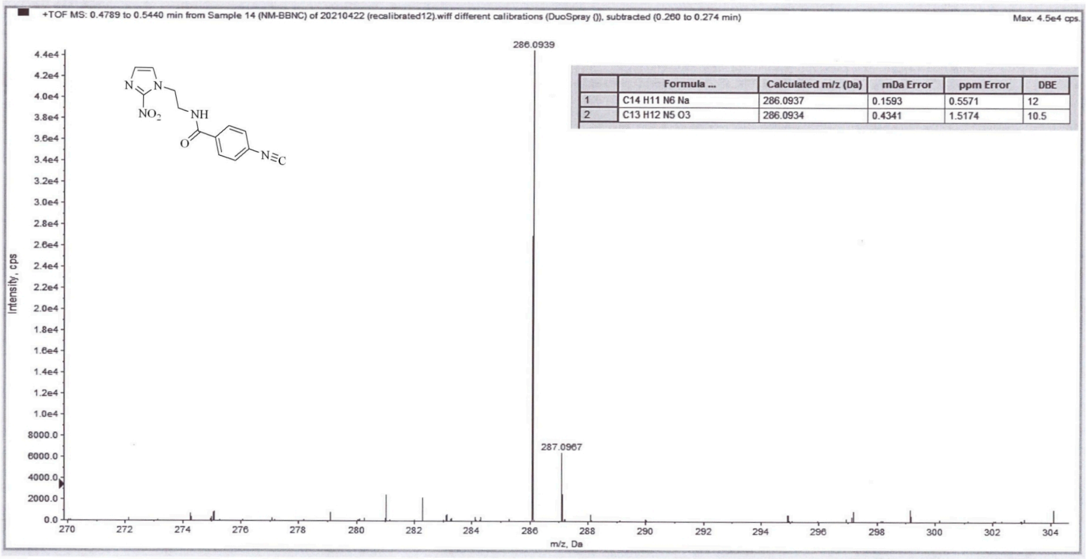

	Formula ...	Calculated m/z (Da)	mDa Error	ppm Error	DBE
1	C14 H11 N6 Na	286.0937	0.1593	0.5571	12
2	C13 H12 N5 O3	286.0934	0.4341	1.5174	10.5

Figure S5. HR-MS chromatogram spectrum of L5.

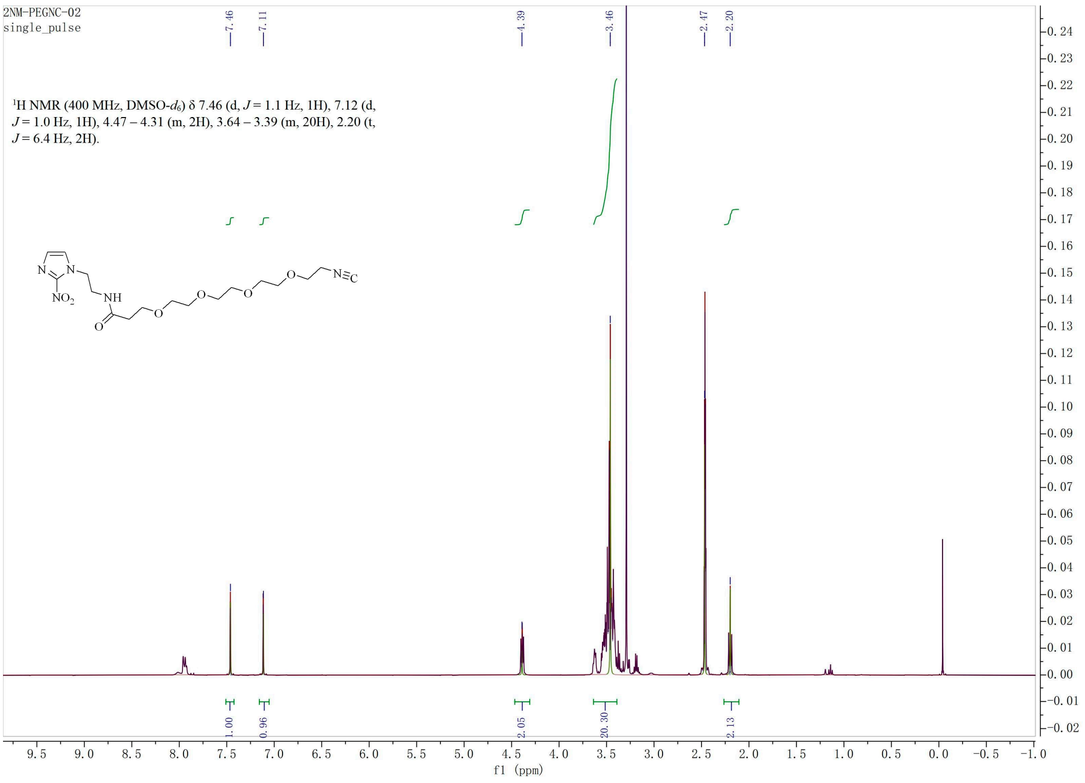

Figure S6. ^{1}H NMR spectrum of L1.

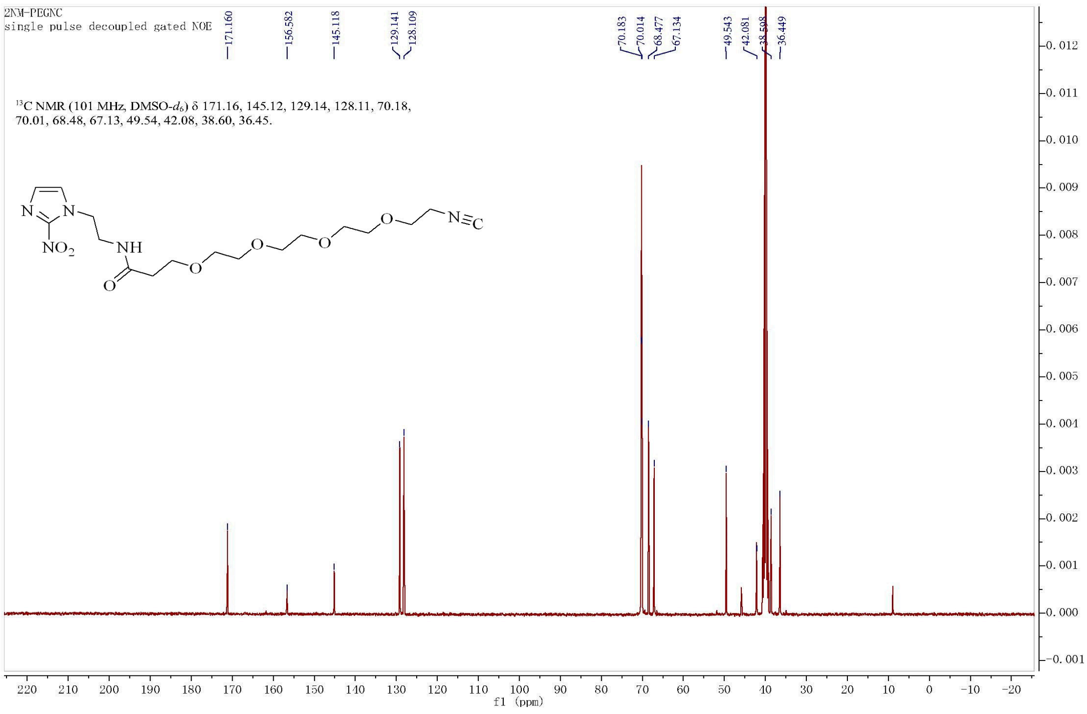

Figure S7. ^{13}C NMR spectrum of L1.

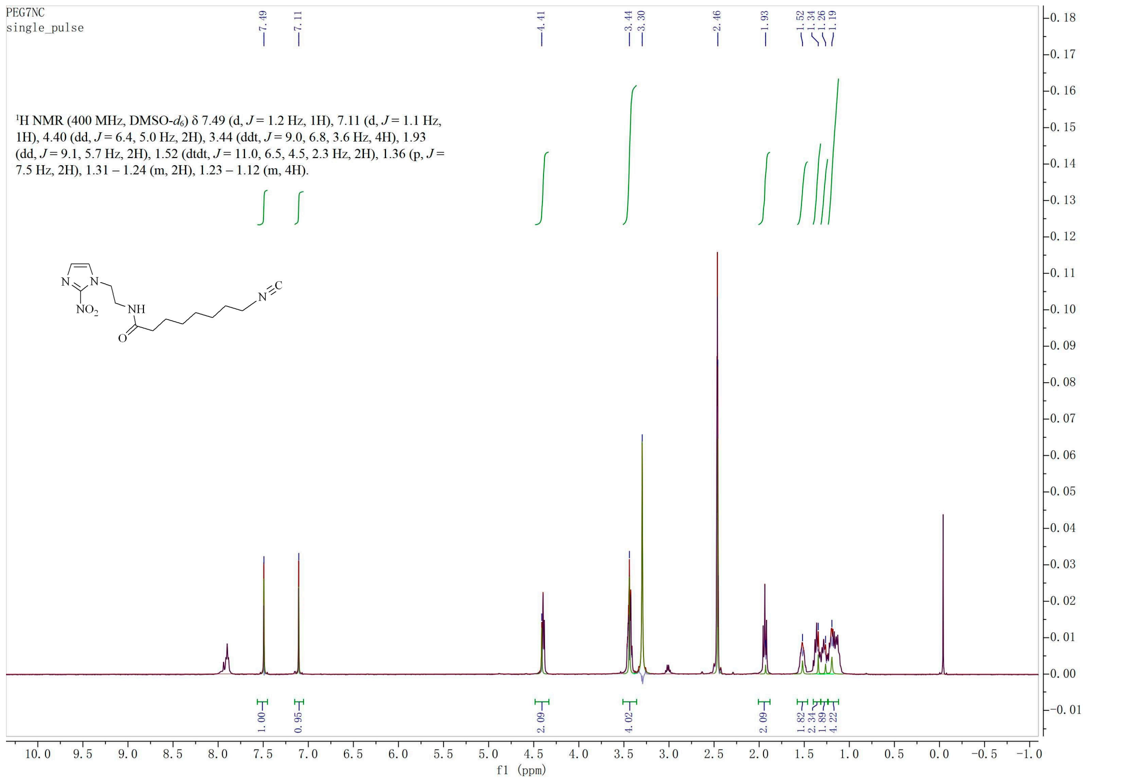

Figure S8. ^{1}H NMR spectrum of L2.

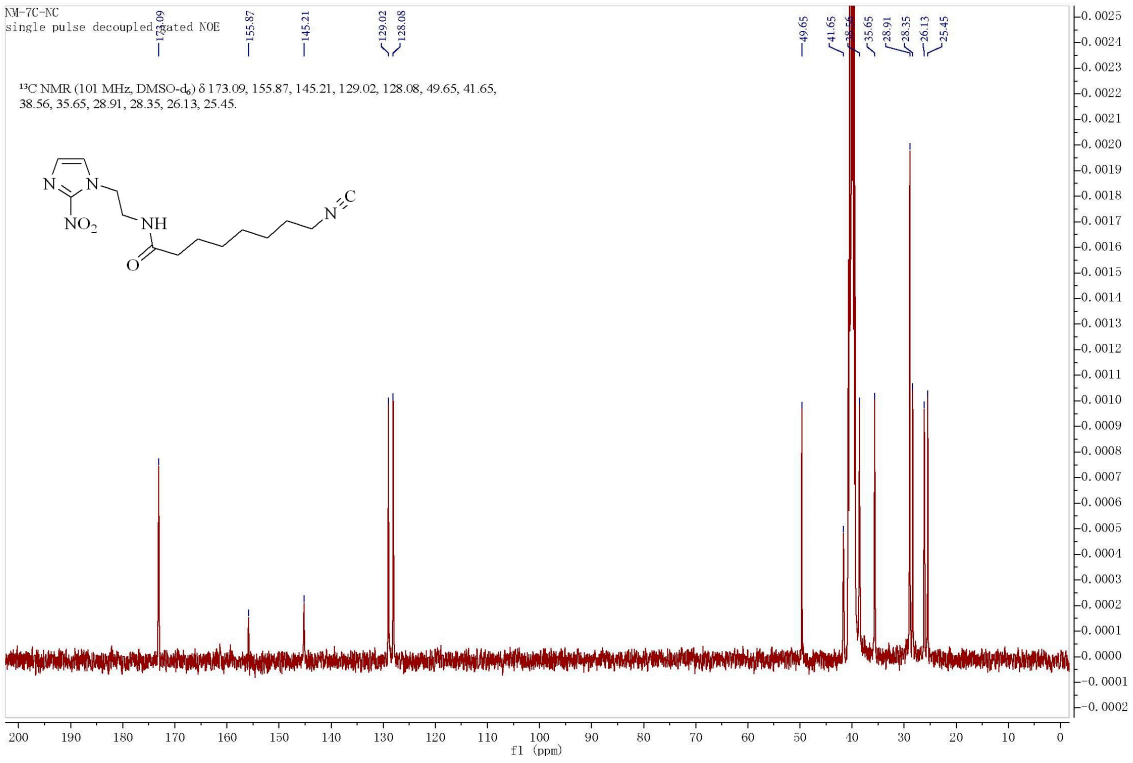

Figure S9. ^{13}C NMR spectrum of L2.

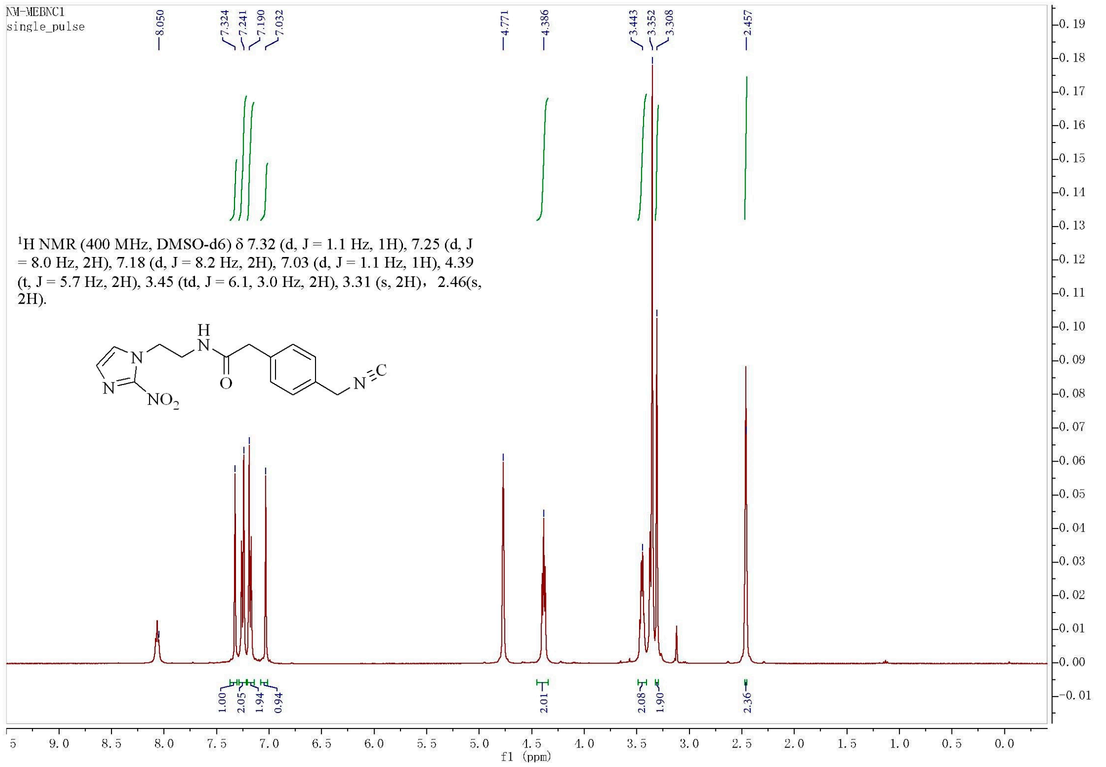

Figure S10. ^{1}H NMR spectrum of L3.

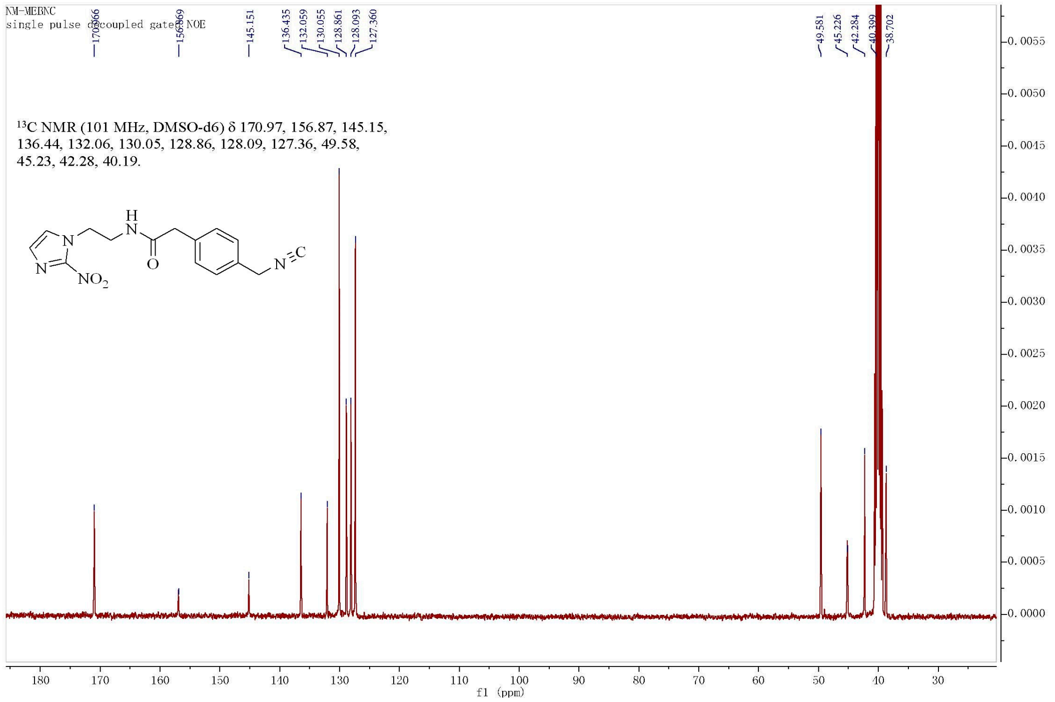

Figure S11. ^{13}C NMR spectrum of L3.

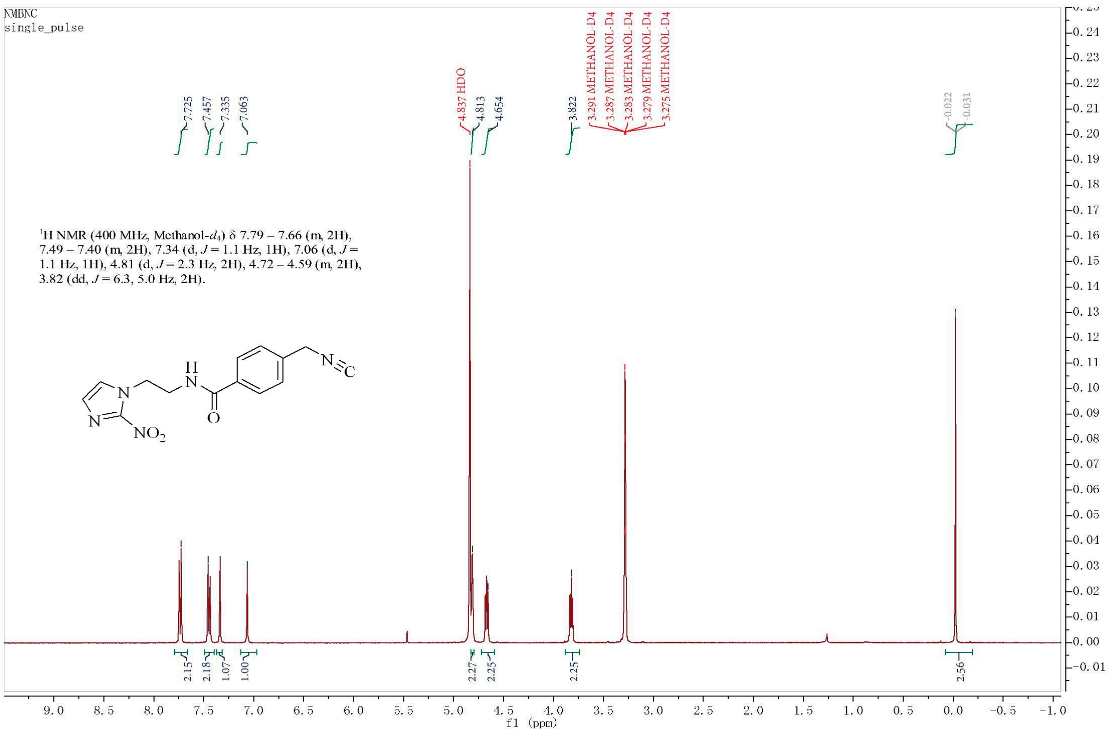

Figure S12. ^{1}H NMR spectrum of L4.

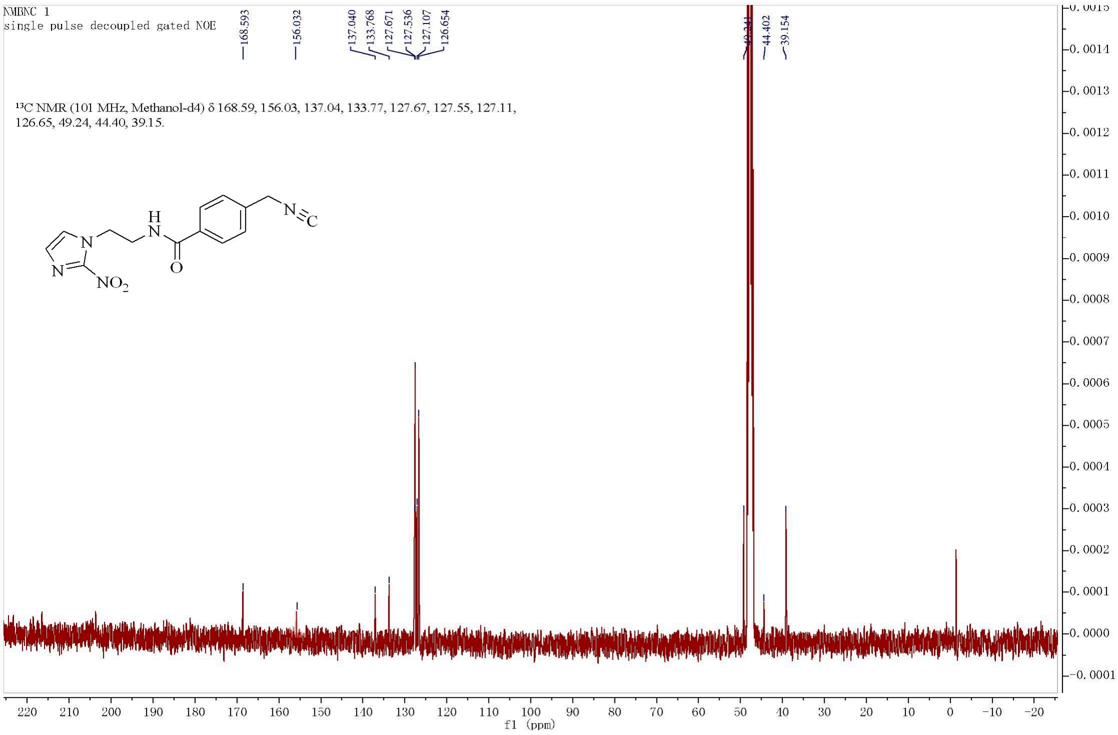

Figure S13. ^{13}C NMR spectrum of L4.

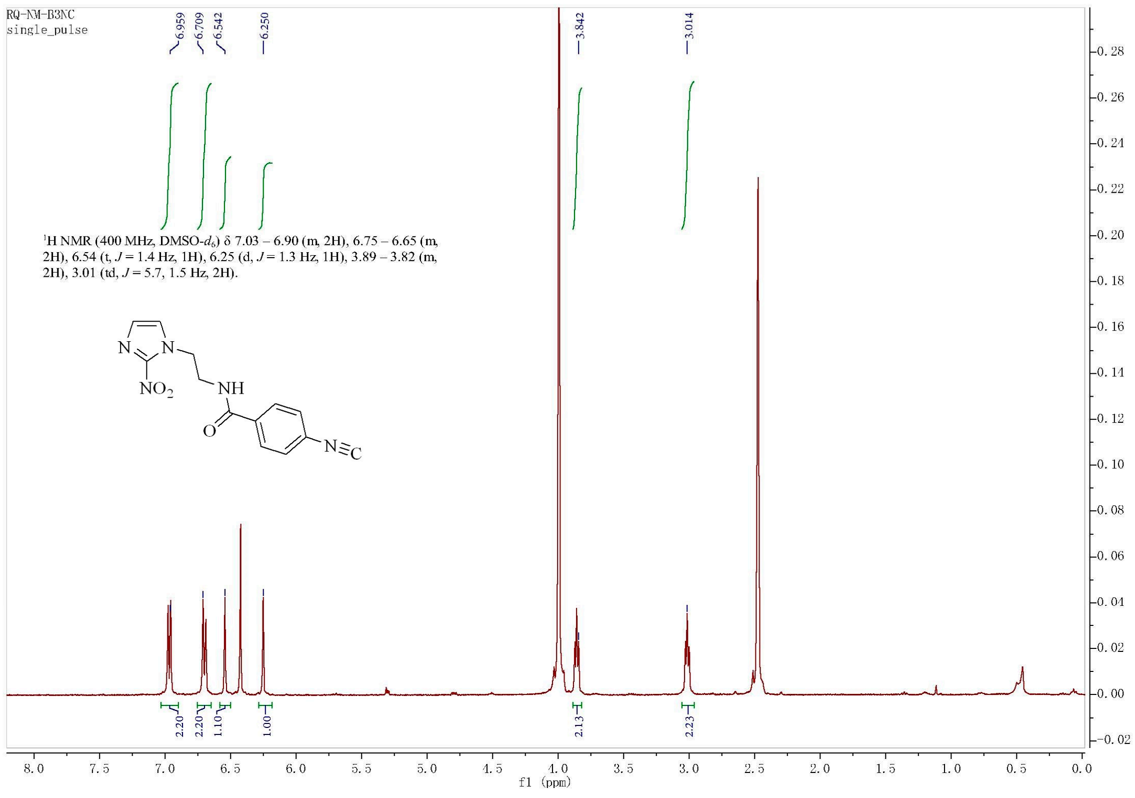

Figure S14. ^{1}H NMR spectrum of L5.

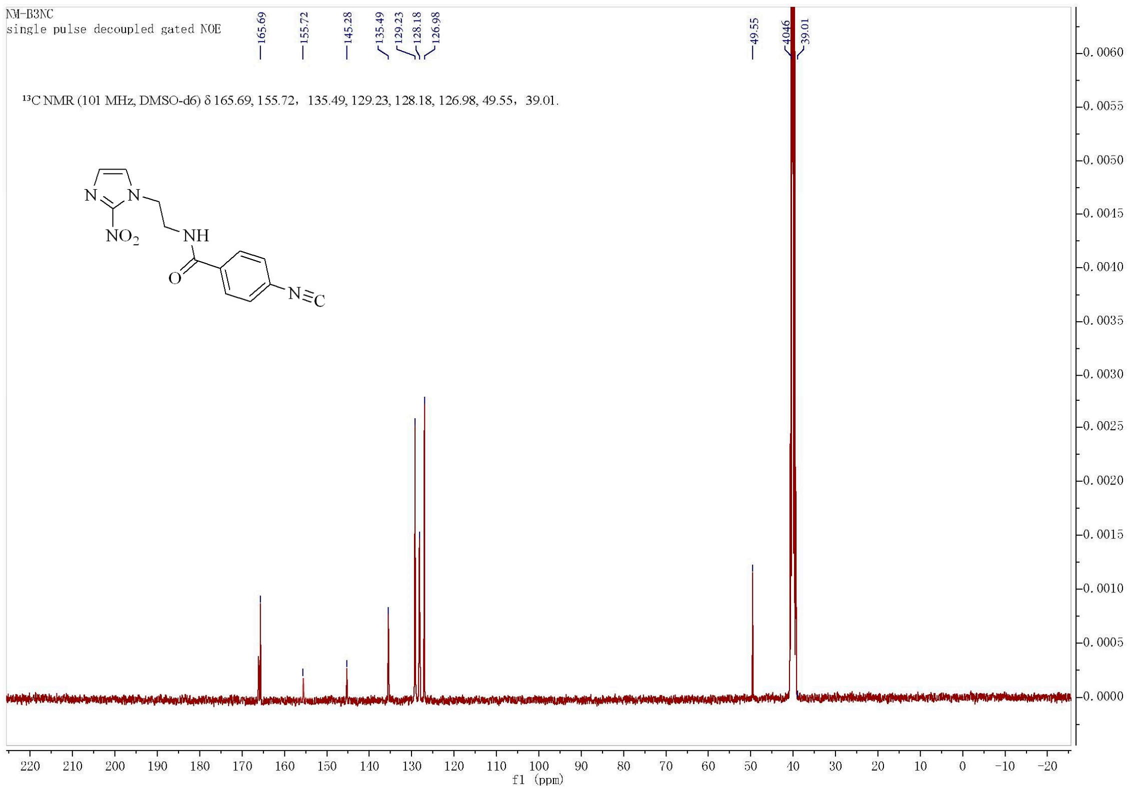

Figure S15. ^{13}C NMR spectrum of L5.

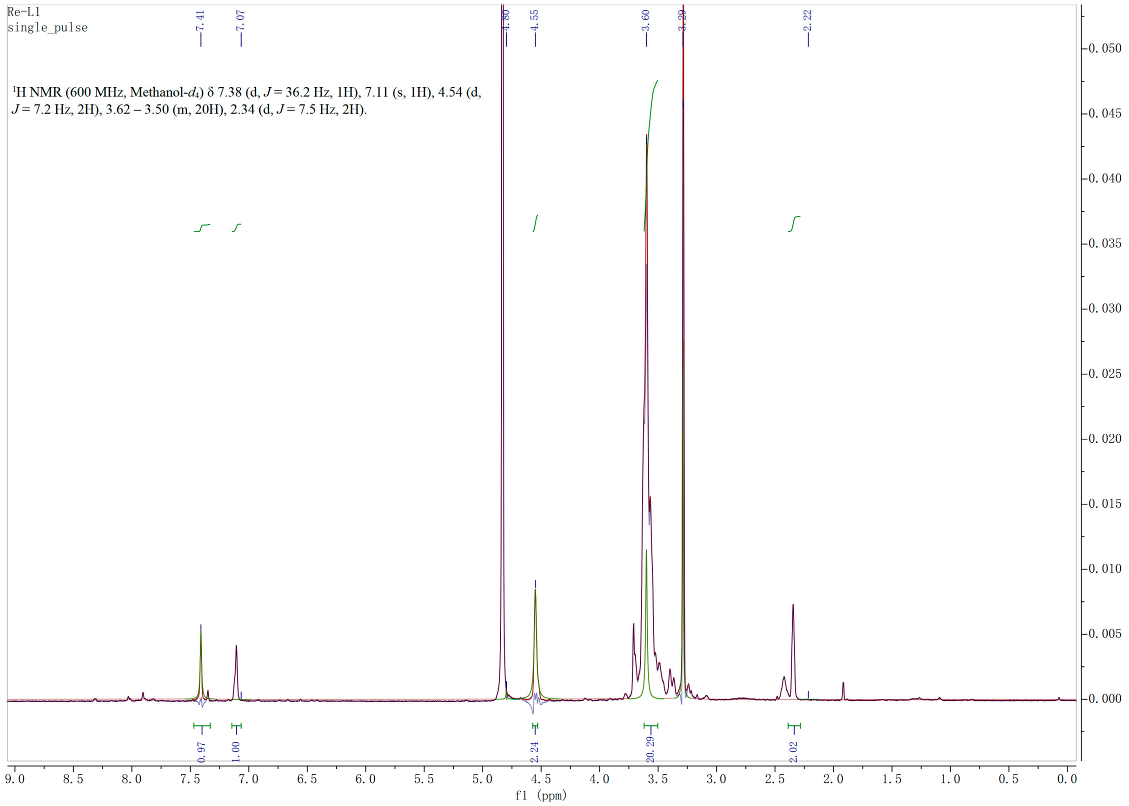

Figure S16. ^{1}H NMR spectrum of Re-L1.

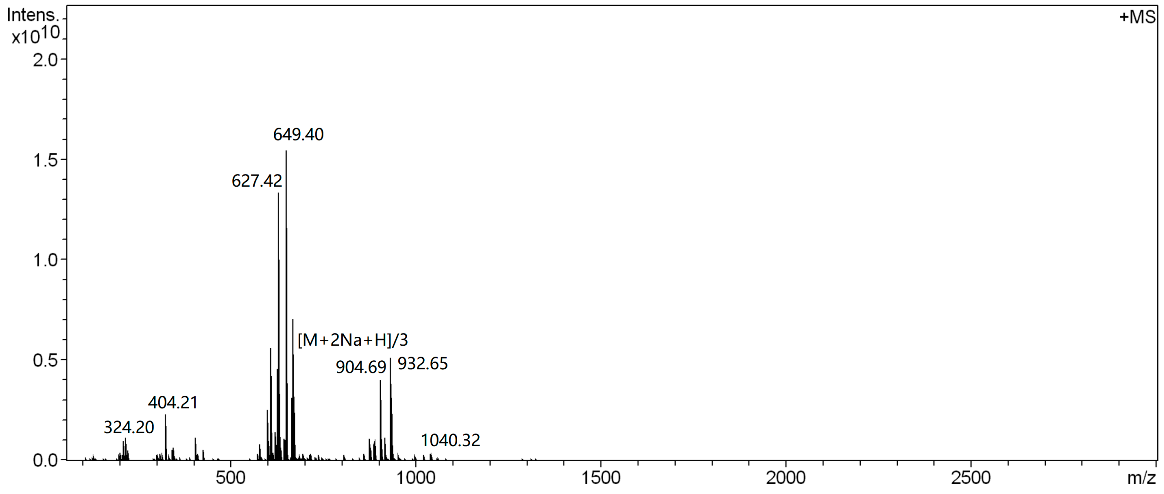

Figure S17. MS chromatogram spectrum of Re-L1.

Table S1. P-values of cellular uptake at different time points.

Complexes	0.5 h	1 h	2 h	4 h
$[^{99m}Tc]$Tc-L1	0.003	0.005	0.006	0.014
$[^{99m}Tc]$Tc-L2	0.004	0.003	0.005	0.007
$[^{99m}Tc]$Tc-L3	0.006	0.009	0.006	0.015
$[^{99m}Tc]$Tc-L4	0.012	0.004	0.021	0.028
$[^{99m}Tc]$Tc-L5	0.021	0.007	0.005	0.005

Table S2. Biodistribution of $[^{99m}Tc]$Tc-L1 in female Kunming mice bearing S180 tumours at 0.5 h, 2 h and 6 h post-injection (%ID/g ± SD**,** n = 5).

	**$[^{99m}Tc]$Tc-L1**		
	0.5 h	2 h	6 h
**Heart**	0.43 ± 0.12	0.12 ± 0.03	0.10 ± 0.03
**Liver**	3.71 ± 0.47	3.49 ± 0.45	2.99 ± 0.17
**Lung**	0.86 ± 0.02	0.24 ± 0.02	0.19 ± 0.05
**Kidneys**	10.08 ± 1.14	8.93 ± 0.80	4.41 ± 0.66
**Spleen**	0.84 ± 0.07	0.65 ± 0.03	0.42 ± 0.07
**Stomach**	0.50 ± 0.08	0.41 ± 0.36	0.41 ± 0.13
**Bone**	0.77 ± 0.09	0.47 ± 0.04	0.34 ± 0.07
**Muscle**	0.32±0.08	0.10 ± 0.04	0.06 ± 0.01
**Small Intestine**	1.34 ± 0.62	0.45 ± 0.15	0.19 ± 0.03
**Large Intestine**	0.71 ± 0.12	2.49 ± 0.20	0.64 ± 0.32
**Tumour**	1.11 ± 0.13	0.47 ± 0.10	0.27 ± 0.05
**Blood**	0.80 ± 0.06	0.13 ± 0.00	0.13 ± 0.03
**Thyroid (%ID)**	0.05 ± 0.02	0.01 ± 0.00	0.02 ± 0.01
**Tumour/Muscle**	4.10 ± 0.63	4.68 ± 0.44	2.18 ± 0.60
**Tumour/Blood**	1.38 ± 0.10	3.81 ± 0.46	4.53 ± 0.75

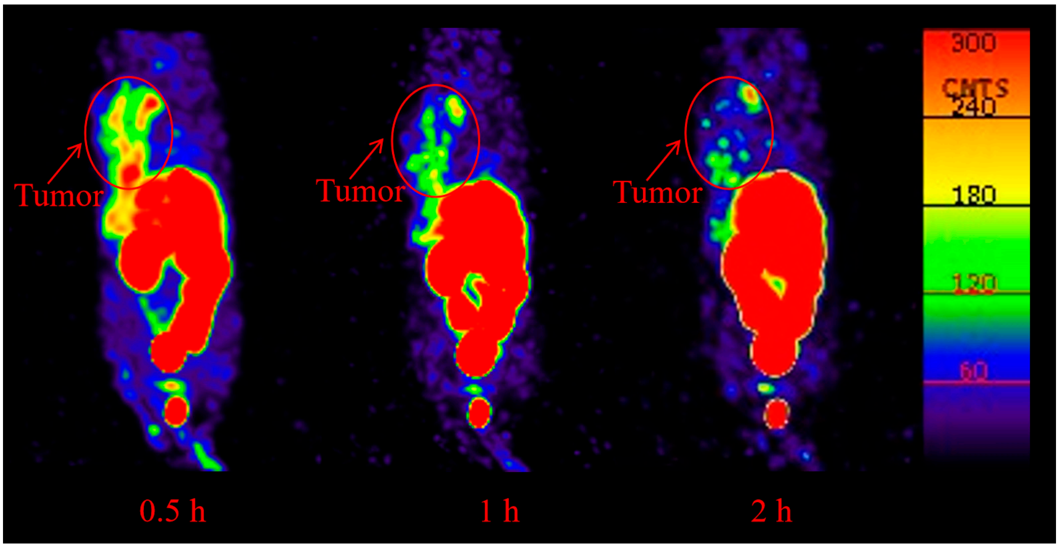

Figure S18. Maximum intensity projection of SPECT images of [^{99m}Tc]Tc-L1 at 0.5 h, 1 h and 2 h post-injection.
